# Genomic, epigenomic, and transcriptomic signatures for telomerase complex components: a pan‐cancer analysis

**DOI:** 10.1002/1878-0261.13324

**Published:** 2022-10-31

**Authors:** Jing Wang, Mingkai Dai, Xiangling Xing, Xing Wang, Xin Qin, Tao Huang, Zhiqing Fang, Yidong Fan, Dawei Xu

**Affiliations:** ^1^ Department of Urologic Oncology, The First Affiliated Hospital of USTC, Division of Life Sciences and Medicine University of Science and Technology of China Hefei China; ^2^ Division of Hematology, Department of Medicine, Bioclinicum and Center for Molecular Medicine Karolinska Institutet and Karolinska University Hospital Solna Stockholm Sweden; ^3^ Department of Urology Surgery The First Affiliated Hospital of USTC, Wannan Medical college Wuhu China; ^4^ Department of Urology Qilu Hospital of Shandong University Jinan China

**Keywords:** cancer, TCAB1, telomerase, telomerase complex, TERC, TERT

## Abstract

Telomerase activation is required for malignant transformation. Recent advances in high‐throughput technologies have enabled the generation of complex datasets, thus providing alternative approaches to exploring telomerase biology more comprehensively, which has proven to be challenging due to the need for laborious assays required to test for telomerase activity. To solve these issues, several groups have analyzed TCGA pan‐cancer tumor datasets by investigating telomerase reverse transcriptase (TERT), the catalytic subunit for telomerase activity, or its surrogates. However, telomerase is a multiunit complex containing not only TERT, but also numerus cofactors required for telomerase function. Here we determined genomic and molecular alterations of 10 well‐characterized telomerase components in the TCGA and CCLE datasets. We calculated a telomerase score (TS) based on their expression profiles and clustered tumors into low, high, and intermediate subtypes. To validate the *in silico* analysis result, we used immunoblotting and telomerase assays. High TS subtypes were significantly associated with stemness, proliferation, epithelial to mesenchymal transition, hyperactivation of oncogenic signaling pathways, shorter patient survival, and infiltration of dysfunctional T‐cells or poor response to immunotherapy. Copy number alterations in 10 telomerase components were widespread and associated with the level of their expression. Surprisingly, primary tumors and cancer cell lines frequently displayed a homozygous deletion of the *TCAB1* gene, encoding a telomerase protein essential for telomerase trafficking, assembling, and function, as previously reported. However, tumors or cells carrying a *TCAB1* deletion still exhibited telomerase activity comparable to or even higher than their wildtype counterparts. Collectively, applying telomerase component‐based TS in complex datasets provided a robust tool for telomerase analyses. Our findings also reveal a tight connection between telomerase and other oncogenic signaling pathways; *TCAB1* may acts as a dispensable telomerase component. Moreover, TS may serve as a useful biomarker to predict patient outcomes and response to immunotherapy.

## Graphical abstract

Comprehensive analyses of TCGA pan‐cancers show widespread genomic and epigenetic alterations coupled with dysregulation in 10 defined telomerase components. The expression‐based telomerase score classification predicts patient survival and treatment to immunotherapy. *TCAB1* gene, encoding a telomerase cofactor essential to telomerase trafficking, assembling, and function, is frequently deleted in different cancer types, but without affecting telomerase activation in tumors.

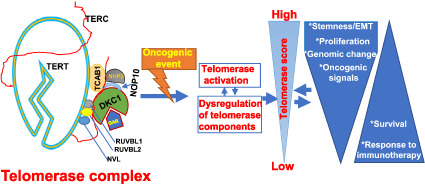

**Abbreviations**
CAF: cancer‐associated fibroblast
CCLE: Cancer Cell Line Encyclopedia
ccRCC: clear cell renal cell carcinoma
CMap: connectivity map
EMT: epithelial to mesenchymal transition
EXTEND: Expression‐based Telomerase ENzymatic activity Detection
FDR: false discovery rate
GSEA: gene set enrichment analysis
HRD: Homologous recombination deficiency
ICB: immune checkpoint blockade
LOH: Loss of heterozygosity
m6A: N6‐methyladenosine
MDSC: myeloid‐derived suppressor cell
NT: normal tissues
OS: overall survival
PFS: progression‐free survival
RSEM: RNA‐Seq by Expectation Maximization
SCNA: somatic copy number alteration
TCGA: The Cancer Genome Atlas
TERC: telomerase RNA component
TERT: telomerase reverse transcriptase
TIDE: Tumor Immune Dysfunction and Exclusion
TMB: Total mutation burden
TPM: transcripts per kilobase million
TRAP: Telomeric repeated amplification protocol
TS: telomerase score

## 1 Introduction

Human linear chromosomes are protected from nucleolytic degradation and illegitimate recombination or fusion by telomeres [1, 2]. Telomeric DNA or TTAGGG repetitive sequences are synthesized by a ribonucleoprotein enzyme called telomerase [3, 4]. In most normal human cells, telomerase is silent, so that telomeres shorten progressively with *in vitro* cell replication or *in vivo* increased age due to the end‐replication problem [1, 5, 6, 7, 8]. When telomere length becomes dysfunctional, the DNA damage response is activated, thereby inducing replicative senescence (permanent growth arrest) or apoptosis of normal cells [1, 5, 9]. Telomere erosion is thus a robust barrier to prevent unlimited cellular proliferation [1, 5, 7, 9]. In contrast to normal somatic cells, human malignant cells are characterized by an immortal phenotype, a key hallmark of cancer [10]. Therefore, such progressive telomere shortening due to telomerase repression serves as a protective mechanism against cancer [6, 7, 8]. During carcinogenesis, overcoming this mechanism by stabilizing telomere length is a prerequisite for malignant transformation of human somatic cells, and it is predominantly achieved by telomerase activation [6, 7, 8, 11, 12]. Indeed, telomerase activity is detectable in up to 90% of human malignancies across cancer types [11, 12].

Telomerase is a multiunit complex, whereas its core enzyme is only composed of a catalytic component telomerase reverse transcriptase (TERT) and internal template‐containing telomerase RNA (TERC) [8]. TERC is almost ubiquitously expressed, while the *TERT* gene is stringently repressed in most normal human somatic cells, which results in telomerase silencing in these cells [6, 7, 8, 13]. Therefore, the transcriptional de‐repression of the *TERT* gene is required for transformed cells to acquire telomerase activity in oncogenesis [6, 7, 8]. Given the key role of TERT in telomerase activation for malignant transformation, great efforts have been made to define various impacts of TERT on cancer development and progression. Numerous studies in the last three decades have significantly contributed to the understanding of the oncogenic effects of TERT and telomerase [6, 7, 8]. Recent advances in next‐generation sequencing and other omics technologies have generated huge amounts of complex datasets, which provide further opportunities to explore TERT and telomerase biology more comprehensively. In this aspect, The Cancer Genome Atlas (TCGA) as a useful resource that provides a large repository of tumor specimens, and several groups have analyzed TERT and telomerase by using this database. In those studies, telomerase activity was estimated based on either TERT mRNA, or TERT‐correlated gene expression and Expression‐based Telomerase ENzymatic activity Detection (EXTEND) [14, 15, 16, 17].

TERT and TERC are sufficient to reconstitute telomerase activity; however, such enzymatic activity is minimal [8]. As described above, telomerase is a multiunit complex with a molecular weight of ~500 kDa, and many other accessory proteins or cofactors in the complex are required for fully functional telomerase [8, 18], whereas the aberrant expression and/or function of these cofactors significantly affects telomerase activity. For instance, DKC1 and its partners NHP2, NOP10, and GAR1, a pseudouridylation enzyme complex stably associated with TERT and TERC, are required for *in vivo* telomerase function, and their mutations or absence leads to diminished telomerase activity and accelerated telomere erosion [18, 19, 20, 21, 22, 23]. TCAB1 has been similarly shown as an essential factor for telomerase activity [19, 24, 25]; however, this gene is next to the tumor suppressor *TP53* on chromosome 17p13.1, a frequently deleted region in cancers [26]. It remains unclear whether telomerase activation is impaired in TCAB1‐deleted tumors. In addition, little is known about the genomic/epigenomic alterations of other telomerase cofactors during the oncogenic process. To thoroughly elucidate telomerase biology in carcinogenesis, it is highly necessary to simultaneously analyze these telomerase components. In the present study, we comprehensively analyzed TERT, TERC, and eight well‐characterized telomerase cofactors for their multidimensional alterations, dysregulation mechanisms, association with oncogenic events, and biological/clinical implications across 33 cancer types derived from the TCGA dataset.

## 2 Materials and methods

### 2.1 Data collection and processing

The TCGA database includes 33 cancer types, and their abbreviations are listed in Table 1. Gene expression (Transcripts Per Kilobase Million, TPM), mutation, and CNA, and clinical information were downloaded from https://gdc.cancer.gov/. The DNA methylation data in TCGA 33 cancer types were collected from http://xena.ucsc.edu/. We obtained expression and CNA information in cell lines from https://sites.broadinstitute.org/ccle/. For clear cell renal cell carcinoma (ccRCC, the predominant subtype of KIRC), patients who received nivolumab or everolimus therapy, transcriptomic, and clinical data were downloaded from the European Genome‐phenome Archive (Accession numbers EGAS00001004290, EGAS00001004291, EGAS00001004292). No ethics approval was required for the present study.

**Table 1:** Summary of TCGA cancer types, abbreviations, and number for telomerase score (TS).

Tumor site	TCGA abbreviation	Number of samples
Adrenocortical carcinoma	ACC	79
Bladder urothelial carcinoma	BLCA	408
Breast invasive carcinoma	BRCA	1103
Cervical squamous cell carcinoma and endocervical adenocarcinoma	CESC	306
Cholangiocarcinoma	CHOL	36
Colon adenocarcinoma	COAD	453
Lymphoid neoplasm diffuse large B‐cell lymphoma	DLBC	48
Esophageal carcinoma	ESCA	185
Glioblastomamultiforme	GBM	168
Head and neck squamous cell carcinoma	HNSC	522
Kidney chromophobe cell carcinoma	KICH	66
Kidney renal clear cell carcinoma	KIRC	534
Kidney renal papillary cell carcinoma	KIRP	291
Acute myeloid leukemia	LAML	173
Brain lower grade glioma	LGG	534
Liver hepatocellular carcinoma	LIHC	374
Lung adenocarcinoma	LUAD	517
Lung squamous cell carcinoma	LUSC	501
Mesothelioma	MESO	87
Ovarian serous cystadenocarcinoma	OV	309
Pancreatic adenocarcinoma	PAAD	179
Pheochromocytoma and paraganglioma	PCPG	184
Prostate adenocarcinoma	PRAD	498
Rectum adenocarcinoma	READ	161
Sarcoma	SARC	263
Skin cutaneous melanoma	SKCM	472
Stomach adenocarcinoma	STAD	415
Testicular germ cell tumors	TGCT	156
Thyroid carcinoma	THCA	513
Thymoma	THYM	120
Uterine corpus endometrial carcinoma	UCEC	533
Uterine carcinosarcoma	UCS	57
Uveal melanoma	UVM	80

### 2.2 mRNA analysis of telomerase components and telomerase score (TS) calculation

TCGA pan‐cancer mRNA data were corrected and standardized for the batch effects. We used the log2 transformed RNA‐Seq by Expectation Maximization (RSEM) data for mRNA analysis and thus log_2_(Fold change) of levels of 10 telomerase components in each cancer type was calculated using the formula: log_2_(mean of tumor) − log_2_(mean of corresponding normal tissues). The difference between them was evaluated using the Wilcoxon signed rank test and the obtained *P* value was adjusted by the false discovery rate (FDR). The TSs for each tumor sample across cancer types were expressed as mean *Z*‐scores based on the *Z*‐normalized mRNA level of 10 telomerase components [27, 28]. mRNA data from CCLE were similarly processed by RSEM normalization. ccRCC mRNA data, from CheckMate 009, CheckMate 010, and CheckMate 025, were log_2_(TPM + 1)‐transformed and batch effect‐corrected using ComBat function by sva package in r (www.bioconductor.org).

### 2.3 Cancer stemness, proliferation, and epithelial to mesenchymal transition (EMT) analysis

The cancer stemness index for TCGA pan‐cancer, which was calculated by correlating the gene weights vector with the gene expression vector per sample using Spearman's correlation, was downloaded from the published study by Malta et al. [29]. Cancer proliferation scores were estimated using levels of Ki67 mRNA. Pan‐cancer EMT scores were calculated by ssGSEA function in the GSVA package using a 16 EMT gene signature as described [30].

### 2.4 Biological/functional differences and 10 oncogenic pathways across three TS‐clusters

We calculated Spearman's correlation coefficients between each expressed gene and TS, ranked them, and performed GSEA analysis using 50 hallmark gene sets [31] to get the normalized enrichment score. *P* values were adjusted by FDR. Pathways with FDR <0.05 in >16 cancer types were selected. For the 10 oncogenic pathways, the chi‐square test was used for comparison between groups, and *P* values were adjusted by FDR.

### 2.5 Genomic instability analysis of tumors

Genomic instability included aneuploidy, somatic total mutation burden (TMB), somatic copy number alterations (SCNA), loss of heterozygosity (LOH), and homologous recombination deficiency (HRD). Their scores were evaluated based on the published system [32]. For aneuploidy, ABSOLUTE and arm‐level clustering algorithms were applied to estimate the total numbers of amplification and deletion at arm‐levels. LOH was directly calculated using ABSOLUTE. Somatic mutation burden represented nonsilent mutations/Mb while somatic CNA burdens were fractions of genome altered or frequency of SCNA segments. The HRD score was calculated using SCNA calls generated from ABSOLUTE together with HRD‐LOH, LST (large‐scale state transitions) and NtAI (number of telomeric allelic imbalances).

### 2.6 SCNA and mutation analysis of telomerase components

TCGA SCNA data per sample was identified by gistic2.0 (http://github.com/broadistitute/gistic2) through ISAR‐corrected Affymetrix genome‐wide human SNP6.0 array data. Amplification, shallow and deep deletion were defined as SCNA value = 2, −1, and −2, respectively. We performed mutation analysis with the following criterions: (a) FILTER defined as “PASS,” “wga,” or “native_wga_mix”; (b) NCALLERS > 1; (c) Variant_Classification defined as “Frame_Shift_Del,” “Frame_Shift_Ins,” “In_Frame_Del,” “In_Frame_Ins,” “Missense_Mutation,” “Nonsense_Mutation,” “Nonstop_Mutation,” “Splice_Site” or “Translation_Start_Site.” CNAs and mutations of telomerase component genes were illustrated using the R package ComplexHeatmap.

### 2.7 DNA methylation and N6‐methyladenosine (m6A) regulator analysis

TCGA DNA methylation data (GDC‐PANCAN.methylation450.tsv.gz) and related files (illuminaMethyl450_hg38_GDC) were downloaded from http://xena.ucsc.edu/. The methylation for the *NOP10* gene was not available. If there were multi‐probes for the same gene, mean β values were used to measure the gene methylation status. For 22 m6A regulators, we calculated coefficients between their mRNA levels and TS using Spearman's correlation.

### 2.8 EXTEND score analysis for telomerase activity

EXpression‐based Telomerase ENzymatic activity Detection (EXTEND) was shown to accurately estimate telomerase activity based on a 13‐gene expression signature (https://github.com/NNoureen/EXTEND) [14]. We applied this approach to analyze telomerase activity in TCGA primary tumors and cancer cell lines from the Cancer Cell Line Encyclopedia (CCLE) dataset.

### 2.9 TIDE score analysis for response to immune checkpoint blockade (ICB)

The Tumor Immune Dysfunction and Exclusion (TIDE) score, a computational framework, has been applied to predict responses to immune checkpoint blockade and determine mechanisms underlying tumor immune escape based on the myeloid‐derived suppressor cell (MDSC), macrophage M2, T‐cell Dysfunction and Exclusion [33]. The TCGA pan‐cancer TIDE score was directly downloaded from http://tide.dfci.harvard.edu/. The TIDE score for the ccRCC cohorts treated with nivolumab was calculated online at http://tide.dfci.harvard.edu/. mRNA expression was standardized by using the all‐sample average expression as the normalization control prior to TIDE score analysis.

### 2.10 Drug sensitivity analysis

We performed drug sensitivity analyses using Connectivity Map (CMap) [34]. CMap is a dataset that contains transcriptomics data from cultured cancer cells treated with >5000 small molecules, which is applied for the discovery of functional connections between drugs, genes, and diseases through the transitory feature of common gene‐expression change. In each cancer type, the expression of the top 150 inversely and positively correlated genes with TS was submitted to https://clue.io/cmap for analyses. Normalized connectivity score (nomalized_cs) was set >1.8 as the threshold. The drugs with normalized_cs >1.8 in more than 15 cancer types were selected.

### 2.11 Cell lines and cell culture

The present study included six cell lines derived from hematological malignancies, K562, HL60, KG1, LP‐1, RPMI8226, and L428. Three of them (K562, RPMI8226, and L428) harbored two *TCAB1* gene copies, while the remaining had *TCAB1* homozygous deletion. K562, HL60, and KG1 cells were from the American Type Culture Collection (Manassas, VA, USA). L428 cells were kindly provided by Prof. V Diehl (University of Cologne, Germany), and LP‐1 and RPMI8226 cells were gifts from Prof. K Nilsson (Uppsala University, Sweden). Cells were incubated in RPMI‐1640 medium (Thermo Fisher Scientific, Waltham, MA, USA) with 10% fetal bovine serum (Thermo Fisher Scientific), 100 U·mL^−1^ penicillin, 100 μg·mL^−1^ streptomycin, and 4 mm L‐glutamine under a humidified 5% CO_2_ atmosphere at 37 °C.

### 2.12 Western blotting

RIPA Lysis Buffer was used for protein extraction from cells. Protein extracts were run on SDS/PAGE and transferred to the PVDF membrane. The membrane was blocked with 5% nonfat milk for 2 h at room temperature and the primary antibodies against TCAB1 (Thermo Fisher) were then added at 4 °C overnight. The secondary antibodies were added the next day; and Bio‐Rad ECL reagents (#1705062) were used to detect proteins on the blots. The ChemiDoc MP Imaging System (Bio‐Rad, Hercules, CA, USA) was used for blot imaging. Actin was used for normalization.

### 2.13 Telomerase activity assay

Proteins were extracted from six cell lines (K562, HL60, KG1, LP‐1, RPMI8226, and L428) and telomerase activity assays were performed using Telomeric repeated amplification protocol (TRAP)‐based TeloTAGGG Telomerase PCR ELISA kit Roche (Merck SA, Darmstadt, Germany) according to the manufactor's protocol. For each assay, 1.0 μg of proteins were used, and 26 PCR cycles were run for amplification. The level of telomerase activity was expressed as arbitrary units based on optimal density absorbance.

### 2.14 Statistical analyses

All statistical analyses were carried out using r package v. 4.0.5 (Vienna, Austria). Comparisons between two groups and among multi groups were made using the Wilcoxon signed rank test and the Kruskal–Wallis test, respectively, and obtained *P* values were adjusted by FDR. Spearman's Rank‐Order Correlation coefficient was applied to determine correlation coefficients R between two variables. The Chi‐squared test was used to evaluate alteration differences in 10 oncogenic pathways among three TS subtypes. The Pearson Correlation coefficient test was to assess the relationship between telomerase activity and TCAB1 protein levels in cell lines. The Survival and Survminer packages were used to make Kaplan–Meier survival curves, and the log‐rank test for statistical analyses. We used the Survival package to evaluate the impact of TS and 10 telomerase components on survival in different cancer types. The European Genome‐phenome Archive (# EGAS00001004290, EGAS00001004291, EGAS00001004292) containing ccRCC (KIRC) patients treated with nivolumab, an antibody blocks the immune checkpoint protein PD‐1, and the MTOR inhibitor everolimus were analyzed for the association between TSs and survival [33]. The treated patients were categorized into high‐ and low‐TS groups based on median TS values.

## 3 Results

### 3.1 Expression profiles of 10 telomerase components across 33 cancer types

**Fig. 1:**
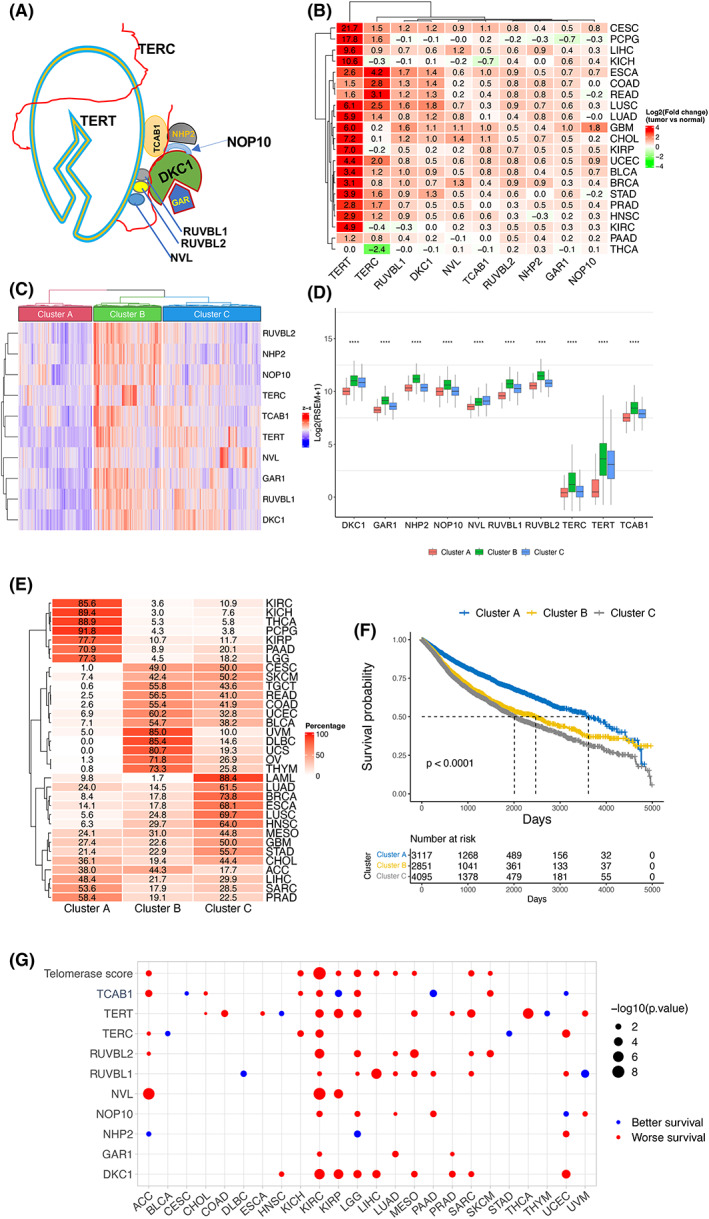
Telomerase component expression‐based stratification of pan‐cancer and association with patient survival. (A) Schematics of the telomerase complex containing 10 telomerase components. (B) Differential mRNA levels of 10 telomerase components between tumors and corresponding adjacent nontumorous tissues (NTs) across cancer types. In 21 of 33 cancer types from the TCGA dataset, ≥3 NTs were available, and shown are expression differences between tumors and NTs. Red and green: higher and lower expression in tumors than in NTs, respectively. (C) The stratification of pan‐cancer according to mRNA levels of 10 telomerase components. Telomerase score (TS), calculated according to telomerase component mRNA levels, was used for unsupervised clustering and three distinct subtypes were revealed: Low (TS‐CA), high (TS‐CB), and intermediate (TS‐CC). (D) Differential mRNA levels of 10 telomerase components in three TS‐subtypes. ****Highly significant differences (*P <* 0.0001) as determined using the Kruskal–Wallis test. (E) Distribution of three TS‐subtypes across cancer types. (F) Association between TS‐subtypes and patient overall survival (OS) in pan‐cancer. (G) Association between OS and TSs or individual telomerase component expression in each cancer type. Blue and red circle: longer and shorter OS in the groups with higher TS or mRNA levels of telomerase components, respectively.

In addition to the core telomerase components TERT and TERC, we included the following telomerase accessory factors for analyses: (a) DKC1, NHP2, NOP10, TCAB1, and GAR1 that are stably associated with the telomerase core TERT and TERC [8], and (b) pontin, reptin, and NVL, three ATPases in the complex providing ATP for telomerase activity [18, 35] (Fig. 1A). We first calculated the differences in expression levels of these 10 genes between tumorous and adjacent normal tissues (NTs) in 21 cancer types, where ≥3 NT samples were available. By doing so, we observed that TERT levels in tumors increased most robustly and widely, which occurred in 20/21 of cancer types except THCA (Fig. 1B). Upregulation of TERC expression was found in 15/21 of cancer types, while a significant reduction was documented in THCA. The level of eight telomerase cofactors was higher in most tumors, but the extent was much less. In cancer types with significant TERT upregulation, levels of other telomerase components in general increased, too (Fig. 1B).

### 3.2 Telomerase component expression‐based stratification of pan‐cancers

The levels of 10 telomerase components were upregulated in most cancer types; however, a significant heterogeneity was observed (Fig. 1B). For comparison, we utilized “telomerase score” (TS) as the overall level of 10 telomerase components according to the expression of these 10 genes in each sample across cancer types. Based on an unsupervised hierarchical clustering in all tumor specimens, we readily identified three distinct clusters: clusters A and B (TS‐CA and TS‐CB), with the lowest and highest expression of TSs or all 10 telomerase genes, respectively, and cluster C (TS‐CC) as the intermediate subtype showing their inconsistent expression levels among 10 genes (Fig. 1C). Further comparisons demonstrated that expression of all 10 telomerase components was highest in the TS‐CB (except NVL), while lowest in the TS‐CA subtype (Fig. 1D). Of note, the vast majority of KIRC, KICH, KIRP, THCA, PCPG, PADD, and LGG, and more than the half of PRAD and SARC, fall into the TS‐CA subtype, while the TS‐CB was predominantly composed of DLBC, UVM, UCS, OV, THYM, and UCEC (Fig. 1E). Most STAD, GBM, HNSC, LUSC, ESCA, BRCA, LUAD, and LAML are in the TS‐CC subtype (Fig. 1E). BLAC, COAD, READ, TGCT, SKCM, and CESC are almost equally distributed between TS‐CB and TS‐CC subtypes, while the number of ACC samples are almost identical in the TS‐CA and TS‐CB clusters (Fig. 1E). TS subtypes of each cancer type are further shown in Fig. S1, which reveal heterogeneity of 10 telomerase components and TS within the same cancer types.

We further evaluated whether these three TS‐clusters could predict patient outcomes. Patient overall survival (OS) was first compared among three subtypes across cancer types. As shown in Fig. 1F, patients in TS‐CA had the longest OS (*P* < 0.0001) compared to those with TS‐CB and TS‐CC subtypes. The impact of TS‐CB and TS‐CC subtypes on OS was similar. Next, comparisons were made within each type of tumor and the significant association between higher TS and worse survival was seen in 10 of 33 cancer types (Fig. 1G). Finally, we determined the effect of 10 individual factors on OS and observed that higher expression of DKC1, NVL, and GAR1 were significantly associated with nine, three, and three cancer types, respectively (Fig. 1G). Higher levels of the remaining seven factors were either positively or negatively associated with OS, depending on tumor types (Fig. 1G). Taken together, a higher TS is the most consistent variable to predict shorter OS.

### 3.3 Correlation of TS with stemness, proliferation, and EMT signatures across cancer types

**Fig. 2:**
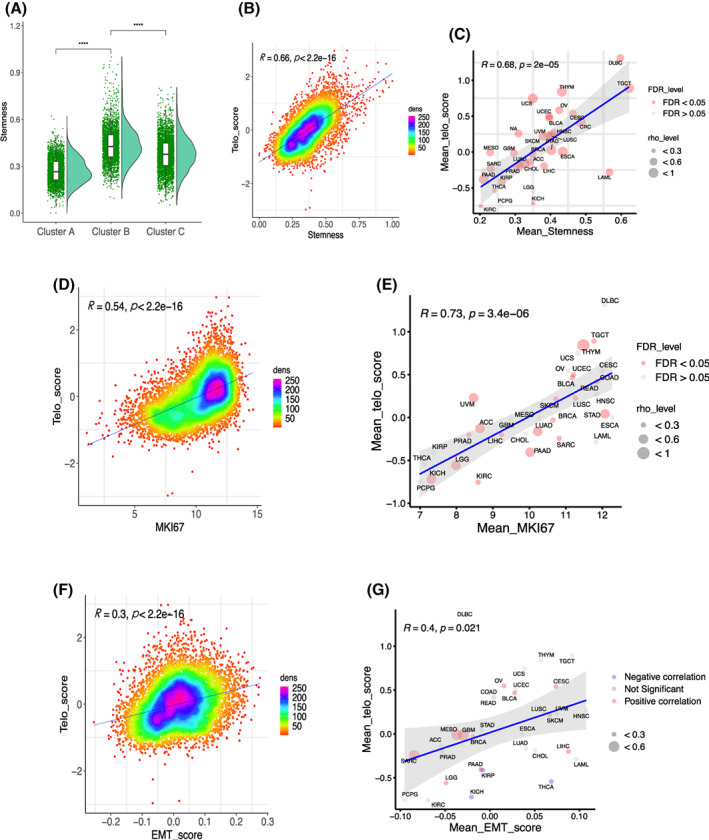
Association of TS‐subtypes with stemness, proliferation, and epithelial to mesenchymal transition (EMT) phenotypes across cancer types. (A) The TS‐CB subtype exhibited the highest stemness score. The analyzed patient number was 9708 and Wilcoxon signed rank test was used. (B) The correlation between TS and stemness score in pan‐cancer. The analyzed patient number was 10 175 and Spearman's Rank‐Order Correlation coefficient was applied. (C) The correlation between TS and stemness score in each cancer type. The analyzed cancer types were 33 and Spearman's Rank‐Order Correlation coefficient was applied. (D) The correlation between TS and proliferation in pan‐cancer. Ki67 expression was used as a proliferation marker. The analyzed patient number was 10 325 and Spearman's Rank‐Order Correlation coefficient was applied. (E) The correlation between TS and proliferation (Ki67 mRNA) in each cancer type. The analyzed cancer types were 33 and Spearman's Rank‐Order Correlation coefficient was applied. (F) The correlation between TS and EMT score in pan‐cancer. The analyzed cancer types were 33 and Spearman's Rank‐Order Correlation coefficient was applied. (G) The correlation between TS and EMT score in each cancer type. The analyzed patient number was 10 325 and Spearman's Rank‐Order Correlation coefficient was applied.

We next sought to determine phenotypic bases of poor patient survival in the groups with higher TSs. Noureen et al. [14] recently showed that telomerase activity estimated using EXTEND was significantly correlated with cancer stemness and proliferation, since these two features in general represent the aggressiveness of tumor behavior. We thus evaluated their relationship with TSs. The cancer stemness index as previously published was used to calculate the stemness score [29], and we observed a robustly positive correlation between TSs and stemness score in total (*R* = 0.66, *P* < 2.2 e‐16), and the TS‐CB subtype had the highest score (Fig. 2A,B). Moreover, a significant correlation was seen in all the cancer types except PCPG and LGG (31/33) (Fig. 2C and Fig. S2). For proliferation, we used Ki67 as a specific marker and analyzed its relationship with TSs. Ki67 mRNA abundance was significantly correlated with TSs across all cancer types (*R* = 0.54, *P* < 2.2 e‐16) (Fig. 2D and Fig. S3). The correlation remained highly significant in most cohorts of each cancer type (*R* = 0.73, *P* = 3.4 e‐06) (Fig. 2E and Fig. S3).

Because cancer stemness is frequently coupled with EMT [36], we further evaluated the relationship between TS and EMT scores. Pan‐cancer EMT scores were calculated using a 16 EMT gene signature, as described [30]. The TS and EMT scores in pan‐cancer were significantly correlated in a positive manner (Fig. 2F). For analyses of individual cancer types, TSs were correlated with EMT scores significantly in 15 types of cancer, while not significantly in 14 cancer types (Fig. 2G and Fig. S4). In four of them (KIRP, KICH, THCA, and PRAD), the inverse correlation was observed (Fig. 2G and Fig. S4).

### 3.4 Differential biological pathway enrichments across the TS‐based subtypes

**Fig. 3:**
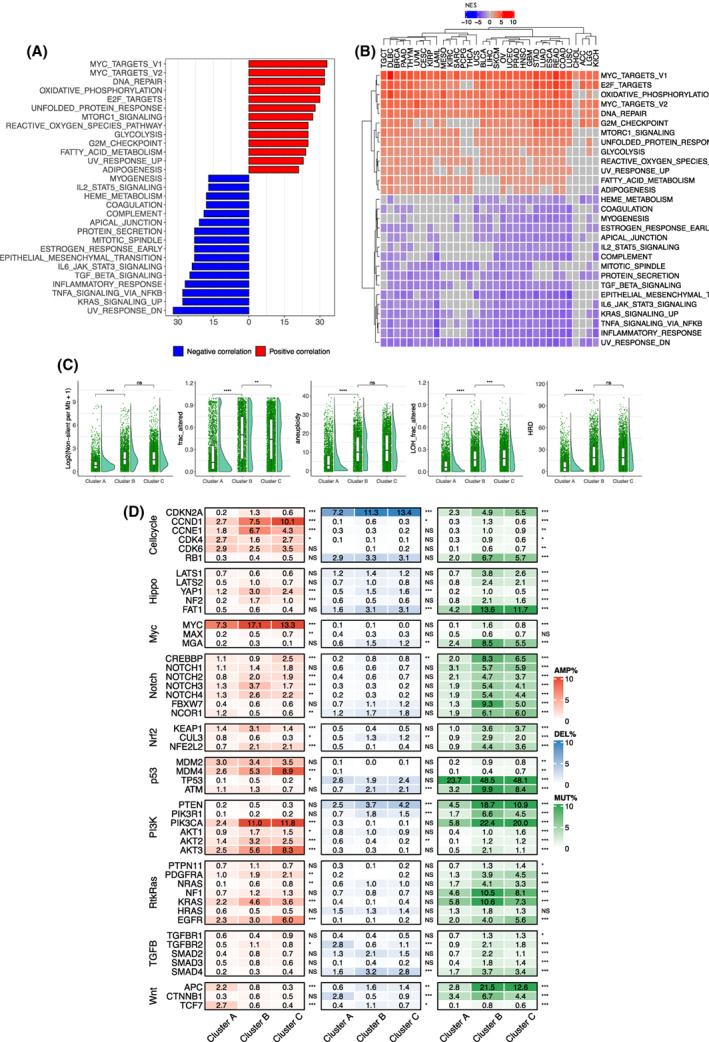
Differences in signaling pathways and genomic alterations among three telomerase score (TS)‐subtypes. (A) Significantly positive or inverse correlation between TSs and signaling pathways across cancer types. The analyzed patient number was 10 325. (B) The correlation between TS and signaling pathways in each cancer type. The analyzed patient number was 10 325. (C) Differences in global genomic alterations among three TS‐subtypes. The analyzed patient number was from 9514 to 9893 and Wilcoxon signed rank test was used. (D) Differences in genomic alterations of 10 common oncogenic signaling pathways among three TS‐subtypes. The analyzed patient numbers were 9991, 9991, and 7170, for amplification, deletion, and mutation, respectively. Chi‐squared test was used. ns, not significant. *, **, ***, and ****: *P* < 0.05, 0.01, 0.001, and 0.0001, respectively.

To characterize biological and molecular differences underlying different phenotypes of three TS subtypes, we performed GSEA analyses. The robustly positive correlation between pathways and TSs included MYC and E2F targets, G2M checkpoint and MTORC1 signaling pathways (Fig. 3A,B). All these enriched pathways mark highly proliferative activity and stemness of cancer cells, which was consistent with the positive correlation between TS and Ki67 amount/stem cell signature observed above. Interestingly, both oxidative phosphorylation and glycolysis pathways were positively correlated with TSs (Fig. 3A,B). Moreover, fatty acid metabolism and adipogenesis pathways were enriched in the higher TS groups. In addition, DNA repair and unfold protein response increased robustly with higher TS in the majority of cancer types. On the other hand, TS was inversely correlated with inflammatory and immune response pathways including TNFα via NF‐κB, IL6‐STAT3, IL2‐STAT5, TGFβ and complement signaling pathways. Unexpectedly, a negative correlation between TS and EMT was observed in 23/33 of cancer types (Fig. 3A,B), which were opposite to those of a positive correlation observed in pan‐cancer analyses (Fig. 2G and Fig. S4).

### 3.5 Featured genomic alterations driving TS‐subtype‐specific activities

The results above reveal biological/molecular differences among three TS subtypes, and we then sought to explore the driving genomic alterations. First, comparisons were made globally: (a) TMB; (b) SCNA; (c) aneuploidy; (d) LOH; and (e) HRD. As shown in Fig. 3C, the TS‐CA subtype exhibited the lowest levels of TMB, SCNA, aneuploidy, LOH, and HRD, while TS‐CB and TS‐CC had robustly higher levels of all these alterations. These aberrations were largely similar to each other or different significantly, but to much less extents between TS‐CB and TS‐CC.

We further focused on the alterations specific to 10 oncogenic pathways, well‐characterized signaling pathways dysregulated across cancer types [37]. Nine of 10 pathways differed significantly across three TS subtypes, while only the TGFβ pathway was largely similar. Among these nine pathways including cell cycle, Hippo, MYC, Notch, NRF2, TP53, PI3K, RTK‐RAS, and WNT, the genomic alterations promoting oncogenic activities while inactivating tumor suppressor functions were much more frequently observed in TS‐CB and TS‐CC than in TS‐CA subtypes (Fig. 3D). For instance, among the PI3K pathway members, mutation/amplification frequencies of the oncogenic PI3KCA were 8.2%, 33.4%, and 31.8% in TS‐CA, TS‐CB, and TS‐CC subtypes, respectively, whereas loss‐of‐function events of PTEN, a negative PI3K regulator, occurred in 7.0%, 22.4%, and 15.1%, respectively (Fig. 3C). Alterations in the cell cycle pathway was highly similar to those in the PI3K members. In the MYC pathway, MYC amplification frequency was more than 2‐fold higher in TS‐CB than in TS‐CA (Fig. 3C).

### 3.6 Functional differences in immune cells among TS subtypes

**Fig. 4:**
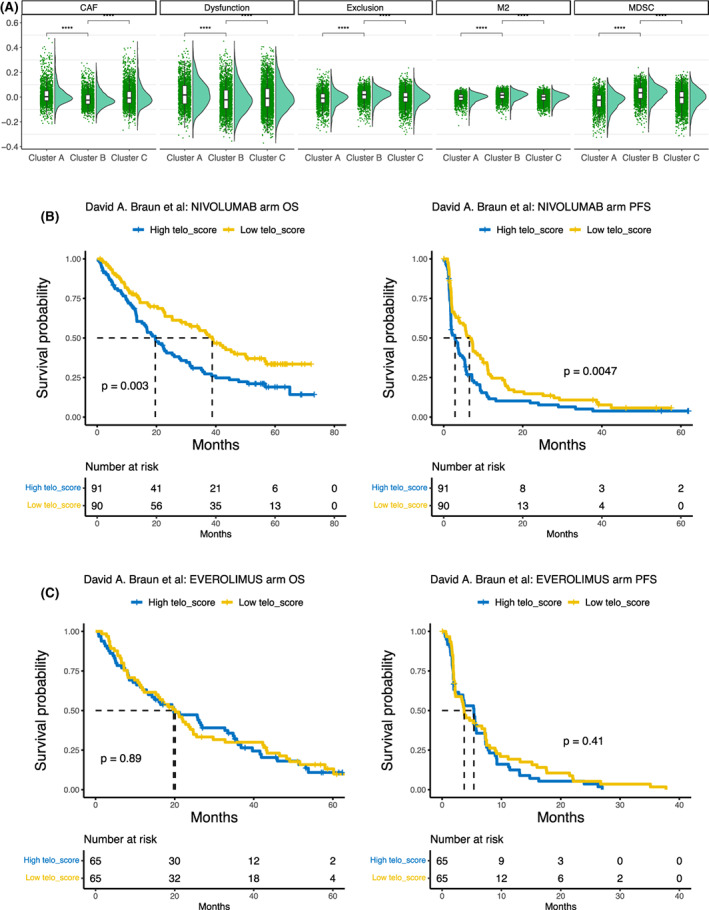
The association between telomerase scores (TSs) and cancer immune escape. (A) Differences in cancer‐associated fibroblast (CAF), myeloid‐derived suppressor cell (MDSC), M2, immune cells among three TS‐subtypes across cancer types, as determined using TIDE (Tumor Immune Dysfunction and Exclusion) analyses. The analyzed patient number was from 7931 and Wilcoxon signed rank test was used. (B) Association between higher TSs and shorter overall survival (OS) or progression‐free survival (PFS) in clear cell renal cell carcinoma (ccRCC) patients receiving PD‐L1 antibody therapy. Log‐rank test was applied. ****P < 0.0001. (C) No association between TS subtypes and OS or PFS in ccRCC patients treated with the MTOR inhibitor everolimus. Log‐rank test was applied.

Because our GSEA analyses showed an inverse correlation between the TS and inflammatory and immune response pathways above, we wanted to probe whether there exist differences in functionality of infiltrated immune cells in tumors across the three TS subtypes. We computed Tumor Immune Dysfunction and Exclusion (TIDE) score [38], a multimetric transcriptional signature shown to better predict immune checkpoint blockade (ICB) response across cancer types. The TS‐CB subtype had the highest myeloid‐derived suppressor cell (MDSC) and M2 macrophage (M2) scores, while the lowest cancer‐associated fibroblast (CAF) score (Fig. 4A). Moreover, the T‐cell exclusion score was highest in TS‐CB. These results suggest that higher TSs promote cancer immune escape. To directly evaluate the influence of TSs on ICB, we analyzed the European Genome‐phenome Archive (# EGAS00001004290, EGAS00001004291, EGAS00001004292) containing ccRCC patients treated with nivolumab, an antibody that blocks the immune checkpoint protein PD‐1 [33]. The treated patients were categorized into high‐ and low‐TS groups according to the tumor median TS value, and the low‐TS group patients had significantly longer OS and PFS (Fig. 4B). In these cohorts, the remaining 130 patients were treated with the MTOR inhibitor everolimus, and we performed the same analysis as above. TSs were associated with neither OS nor PFS in everolimus‐treated patients (Fig. 4C).

### 3.7 Copy number alterations (CNAs) and mutational landscape of the telomerase components across cancer types

**Fig. 5:**
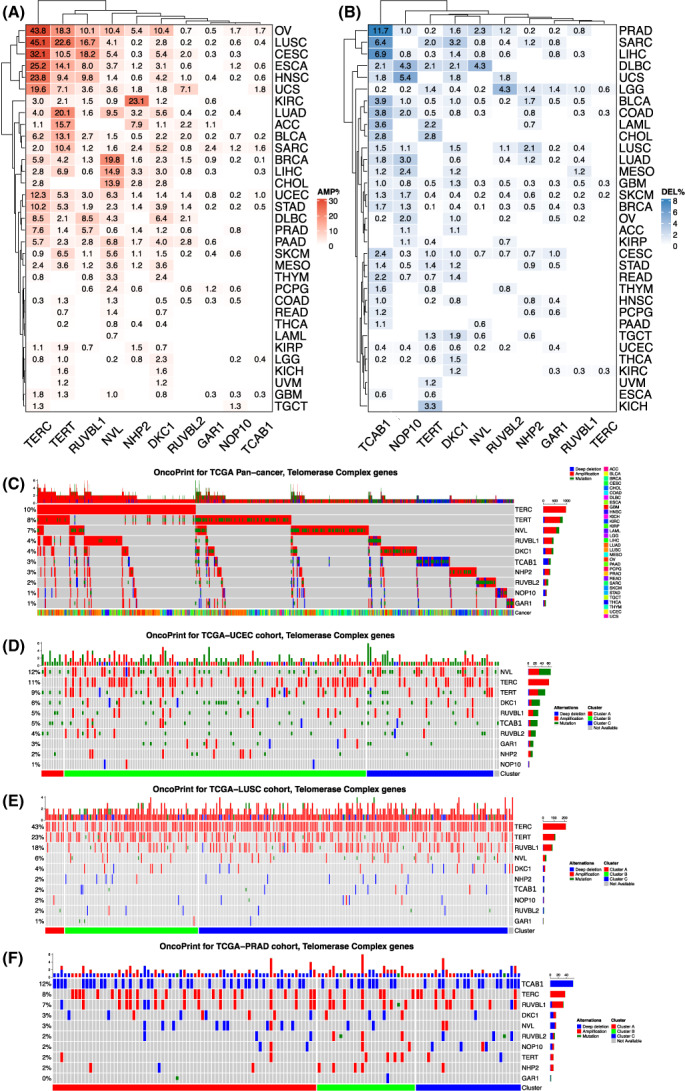
Genomic alterations in 10 telomerase components across cancer types. (A, B) Amplifications and homozygous deletions of 10 telomerase components across cancer types, respectively. The analyzed patient number was 9910. (C) The landscape of overall genomic alterations in 10 telomerase components across 33 cancer types. The analyzed patient number was 10 245. (D–F) Differential genomic alterations of telomerase components in three cancer types. The analyzed patient numbers were 533, 486, and 493 for uterine corpus endometrial carcinoma (UCEC), lung squamous cell carcinoma (LUSC), and prostate adenocarcinoma (PRAD), respectively.

We then sought to investigate potential mechanisms regulating expression of 10 telomerase components. Because previous cytogenetic studies and recent TCGA data analyses have shown a frequent *TERT* and *TERC* gene amplification in various types of cancer [17, 39, 40], we first determined the copy number of 10 telomerase components in 9991 tumor samples from TCGA by calculating amplification and homozygous deletions of these genes. Among 10 telomerase components, the increased *TERC* copy number occurred most, and its amplification was predominant, while homozygous deletion was very rare (Fig. 5A,B). Almost the half of the OV and LUSC, and >1/4 CESC and HNSC tumors harbored *TERC* amplification (Fig. 5A,B). The second most frequently altered gene was TERT, followed by NVL, RUVBL1, and DKC1. Like TERC, the *TERT* amplification was a dominant alteration, but its homozygous deletion was observed in 3.3% of KICH, and ≥2% of CHOL, LAML, DLBC, and SARC tumors (Fig. 5A,B). For NVL2, RUVBL1, and DKC1, the amplification frequency >5% occurred in eight, seven, and five cancer types, respectively. NHP2 amplification was mainly found in KIRC (23.1%), ACC (7.9%), and OV (5.4%). Intriguingly, the homozygous deletion of *TCAB1*, a factor essential to telomerase trafficking, assembling, and function, occurred in 11.7% of PRAD, 6.9% LIHC, and 6.6% SARC tumors (Fig. 5B). For all 10 telomerase components, gene amplification led to the highest levels of their expression (Fig. S5).

We further analyzed somatic mutations of 10 telomerase components based on the nonsynonymous mutation status across 33 cancer types. The total mutation frequency was 4.5% (464/10245 tumors), which mainly occurred in TERT, NVL, RUVBL2, TCAB1, DKC1, and RUVBL1 (Fig. 5C). Mutations seemed random, and at least for TERT and DKC1, no mutations took place in known sites that impair their functions (Fig. 5D and Fig. S6). However, frame‐shift mutations at amino acid 522 of TCAB1 were observed in six tumors (Fig. S6). In addition, the mutational profile of these genes also exhibited the following characteristics: (a) The mutation events were in general mutually exclusive among 10 genes, and (b) The mutation and amplification co‐occurred frequently. On the other hand, mutations of *GAR1*, *NHP2*, and *NOP10* genes were rare, and no *TERC* mutations were documented.

Figure 5D–F further shows featured CNA and mutational signatures of 10 telomerase components in UCEC, PRAD, and LUSC. Both CNAs and mutations of telomerase components were frequent in UCEC, while CNAs were predominant in LUSC and PRAD; however, LUSC and PRAD exhibited widespread amplifications and deletions, respectively.

### 3.8 Telomerase activity in tumor cells bearing homozygous deletions of the *TCAB1* gene

**Fig. 6:**
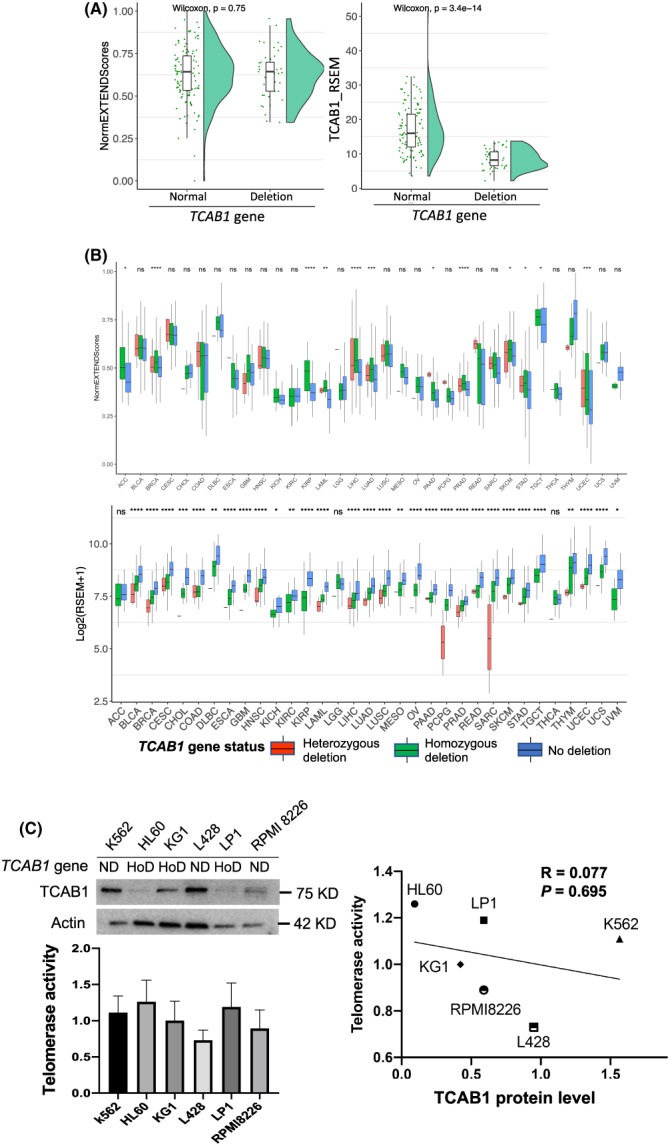
No effects of homozygous *TCAB1* deletion on telomerase activity in cancer cells and primary tumors. (A) Lack of correlation between telomerase activity and *TCAB1* deletion or expression in cancer cell lines. Left panel: Telomerase activity in 164 cell lines derived from hematological malignancies in CCLE was estimated using EXTEND and then compared between cell lines with (45 lines) and without *TCAB1* homozygous deletion (119 lines). Right panel: Significant downregulation of TCAB1 expression in cell lines with *TCAB1* homozygous deletion. (B) Lack of correlation between telomerase activity and *TCAB1* deletion or expression in primary tumors. Top panel: Significant downregulation of TCAB1 expression in tumors with *TCAB1* deletion. The analyzed patient number was 9449. Despite different levels of expression, there was no correlation between telomerase activity and *TCAB1* deletion or expression (Bottom panel). The analyzed patient number was 9996. (C) Telomerase activity in cells with and without *TCAB1* homozygous deletion. Six hematological cancer cell lines were directly analyzed for their TCAB1 and telomerase activity using immunoblotting (Left top) and Telomerase PCR ELISA kit (Left bottom), respectively. Three independent experiments were performed. Right panel: No correlation between telomerase activity and TCAB1 protein levels in six hematological cell lines. Pearson correlation coefficient test was applied. TCAB1 protein quantification was performed using ImageJ based on immunoblotting on the left panel, and arbitrarily expressed as the signal density ratio of TCAB1 and actin. HomoD, homozygous deletion; NA, no alterations; ns, not significant. *, **, ***, and ****: *P* < 0.05, 0.01, 0.001, and 0.0001, respectively.

Because TCBA1 was previously shown to be required for telomerase trafficking, assembling and function [19, 24, 25], we were surprised to find its frequent homozygous deletions in cancer. In the CCLE dataset, 40% of blood cancer‐derived cell lines carry homozygous *TCAB1* deletion, and there was a highly significant reduction of TCAB1 expression in these cell lines (Fig. 6A). Using EXTEND to estimate telomerase activity in these cells, we observed no differences between cell lines with and without *TCAB1* deletion (Fig. 6A). We further evaluated telomerase activity in primary pan‐cancer samples by dividing them into heterozygous, homozygous, deletion, and no deletion (other) of the *TCAB1* gene, and the result showed comparable enzyme activity among the three groups in most cancer types (21/33). In 12 cancer types with significant differences in telomerase activity, the highest EXTEND score was most frequently observed in the group harboring a heterozygous *TCAB1* deletion (Fig. 6B Top), although homozygous and heterozygous deletions of *TCAB1* led to significantly reduced gene expression (Fig. 6B Bottom). We then directly determined telomerase activity in six cell lines derived from hematological malignancies (three myeloid and three lymphoid). HL60, KG1, and LP1 cells carry homozygous *TCAB1* deletions, while the remaining three cell lines do not have its loss. As shown in Fig. 6C, *TCAB1*‐deleted cells expressed significantly lower levels of TCAB1 protein, but their telomerase activity was not decreased or even higher compared with that in cells without *TCAB1* copy deletion. Of note, HL60 cells expressed negligible *TCAB1*, but the highest levels of telomerase activity in all six cell lines (Fig. 6C). There was no correlation between TCAB1 protein levels and telomerase activity in these cell lines (Fig. 6C, right panel).

### 3.9 DNA methylation and m6A regulation of telomerase components

**Fig. 7:**
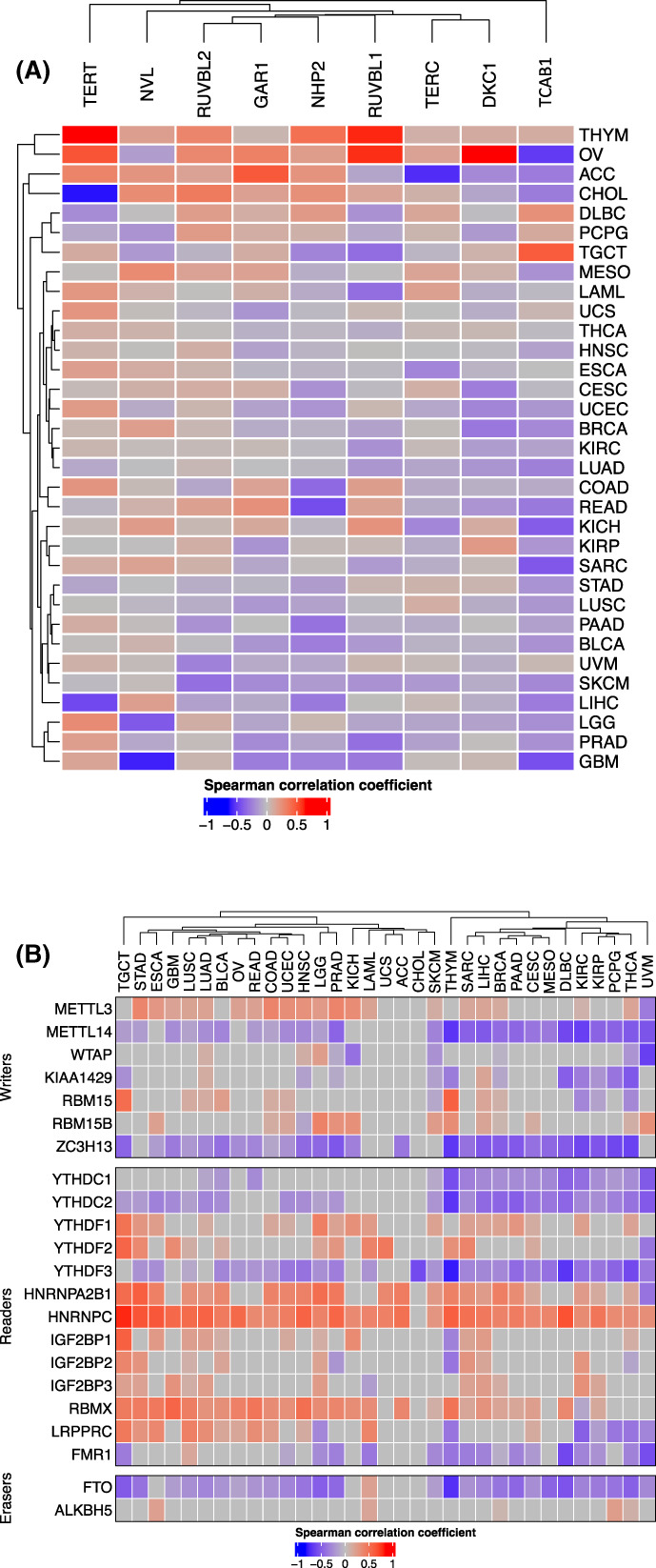
Association of telomerase component expression with m6A regulators and DNA methylation and in pan‐cancer. (A) Differential correlation between m6A regulator and TS. (B) Correlation between DNA methylation and expression of 10 telomerase genes across cancer types.

We further sought to determine the regulatory effect of DNA methylation and m6A on telomerase components. In most cancer types, TERT expression was positively correlated with its locus methylation, which was consistent with previous reports [41, 42]. NVL and RUVBL2 exhibited a similar correlation. For TCAB1, an inverse correlation was observed across almost all cancer types except for TGCT, DLBC, and THYM. DKC1, RUVBL1, NHP2, and TERC expression was also inversely correlated with DNA methylation in most cancer types (Fig. 7A).

Analyses of m6A regulators and TSs showed that TSs were positively correlated with METTL3, while inversely with METTL14 in most cancer types, although both were m6A methyltransferases adding methyl groups to mRNA [43] (Fig. 7B). On the other hand, FTO, a m6A demethylase [43], was inversely correlated with TSs in most cancer types. In addition, m6A‐recruited the binding proteins, YTHDC1, YTHDC2, YTHDF3, and FMR1 were inversely correlated, while HNRNPC, HNRNPA2B1, YTHDF1, and RBMX positively correlated TSs in most cancer types (Fig. 7B).

### 3.10 Drugs targeting telomerase components

**Fig. 8:**
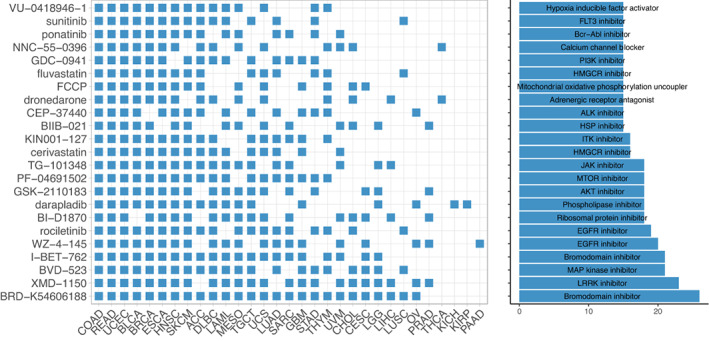
Cancer therapeutic drugs for TS inhibition. Drug sensitivity analyses were performed using Connectivity Map (CMap). Threshold normalized connectivity score (normalized_cs) was set at >1.8. The drugs with normalized_cs >1.8 in more than 15 cancer types passed the sensitivity test.

Telomerase has long been suggested as a cancer‐therapeutic target; however, the development of *in vivo* efficient telomerase inhibitors has only achieved limited success in the last decades. The CMap, a dataset containing a gene expression profile library from more than 5000 small molecule compounds tested in multiple cell types, was used for our analysis. We identified that 23 drugs were inversely correlated with TSs in ≥15 of 33 cancer types. Most of these drugs were inhibitors to target oncogenic signaling pathways. In addition, adrenergic receptor antagonists, calcium channel blockers, mitochondrial oxidative phosphorylation uncoupler, phospholipase, and ribosomal protein inhibitors were inversely correlated with TSs in most cancer types, too. Bromodomain inhibitors, especially the BRD4 inhibitor, displayed the broadest activity, which was consistent with a previous study showing a robust inhibitory effect of BRD4 repression on telomerase activity [44] (Fig. 8).

### 3.11 TS in tumors with alternative telomere‐lengthening (ALT) activation or without telomere maintenance

**Fig. 9:**
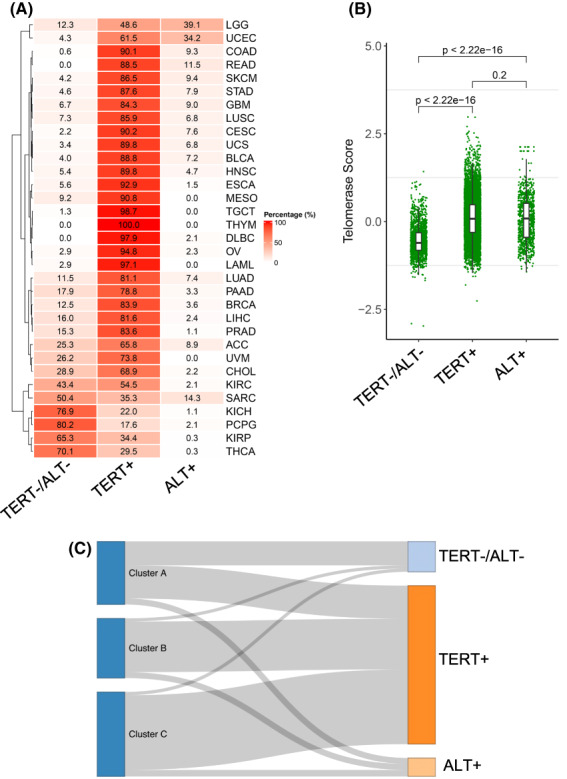
Telomerase score (TS) association with TERT+, alternative telomere lengthening (ALT)+ and TERT−/ALT− tumors across 33 cancer types. (A) Frequencies of TERT+, ALT+, and TERT−/ALT− tumors across 33 cancer types. The analyzed patient number was 11 069. (B) TSs in TERT+, ALT+, and TERT−/ALT− tumors. Wilcoxon signed rank test was applied. TERT−/ALT− *vs* TERT+ and TERT−/ALT− *vs* ALT+, *P* < 2.2 e‐10^16^, and TERT+ *vs* ALT+, *P* = 0.20. (C) The Sankey diagram showing the TS subtype distribution among TERT+ (8110), ALT+ (949), and TERT−/ALT− (1557) tumors.

The telomerase‐mediated telomere extension is the predominant mechanism in cancer, while the ALT pathway is activated in a small fraction of human malignancies [17]. More recently, aggressive tumors without telomere maintenance have been also observed [45, 46]. To make comparisons, we sorted out ALT‐tumors based on the *ATRX* or *DAXX* gene mutations, TERT‐positive tumors, and double negative (TERT−/ALT−) tumors. In a total of 11 352 tumors from TCGA, we observed that TERT+, ALT+, and TERT−/ALT− tumors were 8424 (74.2%), 949 (8.4%), and 1979 (17.4%), respectively. Figure 9A shows the distribution of three different tumors across 33 cancer types. TSs in TERT−/ALT− tumors were the lowest, while highest in TERT+ tumors, and statistical differences were observed between TERT−/ALT− and TERT+ tumors or ALT+ tumors (Fig. 9B). TSs were higher in TERT+ tumors than in ALT+ tumors, but the difference was not statistically significant (Fig. 9B). The Sankey diagram further showed the relationship between these tumors and TS subtypes: the TS‐CB and CC subtypes were mainly derived from TERT+ tumors, and TS‐CA was from both TERT+ and TERT−/ALT− tumors, whereas ALT+ tumors were evenly distributed among three TS subtypes (Fig. 9C).

## 4 Discussion

Several recent studies have analyzed TERT alterations and/or telomeres/shelterin in TCGA pan‐cancers, gaining broader insights into telomerase regulation and the role in carcinogenesis [14, 15, 16, 17, 47]. However, the fully functional telomerase needs not only its core component TERT, but also TERC and accessory proteins [8]. In the study presented herein, we determined the genomic and epigenomic alterations of 10 important telomerase components across 33 cancer types with >10 000 samples, which provides a comprehensive landscape of their aberrations or dysregulations, and their association with different oncogenic signaling pathways, cancer hallmarks, and clinical variables.

Telomere maintenance in cancer is predominantly achieved by telomerase activation, and telomerase activity is detectable in up to 90% of human malignancies [12]. Consistently, the present findings reveal a widespread dysregulation of telomerase components; however, a significant expression heterogeneity of 10 telomerase components was observed across and within cancer types. Based on their expression profile or TS, all TCGA pan‐tumors could be largely classified into three subtypes: low, high, and intermediate. We observed that prevalent CNAs in these 10 telomerase components, and copy number gains or amplifications significantly contribute to upregulation of their expression, especially for TERT and TERC. Intriguingly, however, *TCAB1* gene neighbors with *TP53* on chromosome 17 [26], and the simultaneous loss of both genes, are observed in 26 of 32 cancer types. Homozygous deletion of *TCAB1* occurs most frequently in primary PRAD tumors (11.7%). Cancer‐derived cell lines exhibit a much higher frequency of the *TCAB1* deletion, with 40% in cell lines derived from hematological malignancies. In addition, frame‐shift mutations at a single site (amino acid 522) of the TCAB1 protein were observed in six tumors. It was previously shown that TCAB1 was required for telomerase assembling, trafficking, and enzyme activity, and TCAB1‐KO HeLa and normal stem cells exhibited an 80% reduction in telomerase activity [19, 24, 25]. Heterozygous *TCAB1* missense mutations were shown to impair telomerase trafficking, causing dyskeratosis congenita due to defective telomere maintenance [21]. These observations thus raise a critical question of how telomerase activity is maintained in TCAB1‐null cancers. Using EXTEND to estimate telomerase activity, we did not observe significant differences in either primary tumors and cell lines with and without *TCAB1* deletions. Moreover, we directly assessed telomerase activity in malignant cells, and the results demonstrated that high levels of telomerase activity were readily detected in *TCAB1*‐null cells, which was comparable to those in cells harboring two copies of *TCAB1*. These findings show that TCAB1 insufficiency or even deficiency does not lead to inhibition of telomerase activity and of telomere stabilization at least in cancer cell lines. It is currently unclear how *TCAB1*‐deficient cancer cells maintain their telomerase activity, or whether the function of TCAB1 is compensated by other factors. Further investigations are required to answer these important questions.

In addition to CNAs, epigenetic factors including m6A regulators and DNA methylation might be actively involved in the regulation of telomerase component expression. We observed the significant correlation between TSs and several m6A modifiers. The m6A demethylase FTO was inversely correlated with TSs in most cancer type, suggesting that the RNA demethylation inhibits expression of telomerase components. On the other hand, TSs were positively correlated with METTL3, consistent with the FTO effect. However, METTL14, another m6A writer, was inversely correlated with TSs in most cancer types, which is not Consistent with the suggestion above. These issues await elucidation using cellular molecular approaches. For DNA methylation, TERT expression is in general positively correlated with hypomethylation, in accordance with previous reports [42]. Interestingly, the expression and DNA methylation for the *TCAB1* gene was inversely correlated in most cancer types. Likely, the aberrant methylation at *TCAB1* loci leads to its downregulation.

Our comprehensive analyses further showed that TSs were significantly associated with patient OS, and the low TS cluster TS‐CA had the longest OS, while high TS‐CB predicted shorter OS. Consistently, TS‐CA tumors exhibited the lowest stemness and proliferation potential and were associated with minimal levels of global genomic alterations including TMB, LOH, aneuploidy, and CNAs. Further characterization of 10 oncogenic signaling pathways commonly dysregulated across cancer types reveals significant differences in 9 of 10 across three TS subtypes. For these nine signaling pathways, including cell cycle, Hippo, MYC, Notch, NRF2, TP53, PI3K, RTK‐RAS, and WNT, the genomic alterations promoting oncogenic activities while inactivating tumor suppressor functions were much more frequently observed in TS‐CB and TS‐CC than in TS‐CA. Among the PI3K pathway members, mutation/amplification frequencies of the oncogenic PI3KCA were almost 4‐fold higher in TS‐CB and TS‐CC than in TS‐CA subtypes, whereas loss‐of‐function events of PTEN, a negative PI3K regulator, occurred 2–3‐fold higher in TS‐CB and TS‐CC subtypes. On the other hand, the GSEA analysis revealed highly positive correlations between TSs and signaling pathways including MYC and E2F targets, G2M checkpoints, MTORC1, oxidative phosphorylation and glycolysis, fatty acid metabolism, and adipogenesis. All these enriched pathways in the higher TS groups are consistent with observed active proliferation and stemness phenotypes. Interestingly, the identified drugs most efficiently repressing TS are inhibitors for BRD4, EGFR, MAPK, PI3K, AKT, JAK, ITK, FLT3, etc., and almost all of these targets are in the higher TS subtypes.

The correlation analysis showed a significantly positive correlation between TS and EMT scores in 15 cancer types; however, the inverse correlation between them was observed in 23 cancer types as determined by GSEA. For the correlation analysis, a 16 gene signature including only the key EMT factors/markers was applied, while there were hundreds of EMT‐related genes in the GSEA analysis and the GSEA assessment of an EMT phenotype may thus be less specific, which provides a potential explanation for the inconsistent results between these two different analyses. Given the hyperactive oncogenic signaling pathways in higher TS tumors, it is conceivable that increased TSs promote EMT. It is also likely that the TS effect on EMT is context‐ or cancer type‐dependent.

Our findings also provide insights into the relationship between TSs and tumor stroma including immune cells. The TS‐CS subtype had the highest MDSC and M2 scores, while MDSC and M2 enrichments in the tumor microenvironment either prevent infiltration or lead to dysfunction of cytotoxic T‐cells [38]. Indeed, the T‐cell exclusion score was the highest in TS‐CB. These results suggest that higher TSs promote cancer immune escape. Our analyses of advanced ccRCC (KIRC) patients treated with the PD‐1 antibody nivolumab [33] showed that patients with low TS‐tumors had significantly longer OS and PFS. In this cohort of patients, conventional genomic markers including tumor mutation burden and neoantigen load failed to predict nivolumab response, while the PBRM1 truncated mutation was associated with longer patients OS and PFS [33]. Both low TS and PBRM1 truncated mutation exhibited comparable prediction capacity. Thus, TS may serve as a useful predictor for ICB response. On the other hand, OS and PFS were not associated with TSs in ccRCC patients treated with the MTOR inhibitor, further suggesting the specific impact of TS on ICB therapy. Further studies are required to validate our proof‐of‐concept observations.

Our analyses of the TCGA tumors revealed that 17% of them lack TERT expression and *ATRX* or *DAXX* mutations, indicating that telomere maintenance is absent in these tumors. Barthel et al. [17] previously analyzed the TCGA pan‐cancers and identified that telomerase+ and ALT+ tumors were 73% and 5%, respectively, while the remaining 22% were negative for both, which was largely consistent with the present result. It should be recognized that RNA seq was applied for transcriptomic analysis in TCGA tumors, and its sensitivity might be insufficient to detect low levels of TERT mRNA, which resulted in underestimation of TERT+ tumors. For instance, the frequency of TERT+ tumors in the TCGA THCA cohort (papillary subtype predominant) was only 30% [48]; however, the PCR‐based assay in general showed that 40–60% of them were TERT‐positive [49, 50, 51]. Unexpectedly, we did not observe a significant difference in TSs between TERT+ and ALT+ tumors. ALT+ tumors do not express TERT but may have similar or even higher levels of the remaining nine telomerase components, which thus maintains overall levels of TSs. Interestingly, TERT−/ALT− tumors exhibited the lowest mRNA levels of 10 telomerase components. Recent studies have shown that some aggressive melanoma and neuroblastoma‐derived cells lack telomere maintenance [45, 46]. It is currently unclear how these tumors are formed and progressed *in vivo*, which is worthy of detailed investigations.

It should be pointed out that all these telomerase components exhibit many different biological activities in addition to their role in telomerase function. For example, TERT serves as a transcriptional cofactor to regulate expression of a panel of oncogenic factors, and promotes cancer aggressiveness, resistance to oxidative stress, and anticancer drugs, and survival or proliferation [52, 53, 54, 55, 56, 57, 58, 59, 60, 61]. TERC is associated with not only telomeres but also acts as a transcription factor to regulate the transcription of WNT, inflammation‐related, and myeloid genes [62, 63, 64]. In addition, the DKC1/NOP10/NHP2 complex‐mediated pseudouridylation broadly regulates RNA metabolism, including RNA stability, ribosome biogenesis, and splicing events [20, 65]. Therefore, their effects may be beyond telomere lengthening in cancer cells. The present findings reveal a tight correlation between TS and aggressive phenotypes, and significant impacts of TSs on tumor stroma and response to immunotherapy. Moreover, genomic aberrations, tumor suppressor inactivation, and oncogene activation are robustly enriched in TS‐high tumors. These TS‐related alterations are likely telomere lengthening‐independent, supporting the noncanonical role of telomerase in oncogenesis.

## 5 Conclusion

In summary, we directly analyzed 10 telomerase components across human cancer in the present study and identified three distinct subtypes based on their expression heterogeneity. Differential expression of telomerase components is attributable to their CNAs and epigenetic aberrations. One of the key findings is that high telomerase activity is present in TCAB1‐deficient cancer cells, which is inconsistent with previous reports [24, 25]. Importantly, three different subtypes are differentially associated with cancer stemness, EMT, proliferation, metabolic reprograming, genomic instability, immune escape, and patient survival driven by various oncogenic signaling pathways. Clearly, telomere maintenance is integrated into the whole oncogenic program during carcinogenesis and targeting upstream events may help to efficiently block telomere‐related aberrations in cancer.

## Conflict of interest

The authors declare no conflicts of interest.

## Author contributions

JW, ZF, YF, and DX designed the study. MD and XX carried out the experiments. JW, MD, XQ, XW, TH, and ZF performed bioinformatic analyses. ZF, YF, and DX supervised the research works. JW, MD, ZF, YF, and DX wrote and revised the article. All authors reviewed and edited the article. All authors read and approved the final article.

## Supporting information


**Fig. S1.** Telomerase score (TS) and distribution of the three TS subtypes in each cancer type.
**Fig. S2.** Correlation between telomerase score (TS) and stemness in each cancer across 33 cancer types.
**Fig. S3.** The correlation between telomerase score (TS) and proliferation marker KI67 level in each cancer type.
**Fig. S4.** The correlation between telomerase score (TS) and EMT score in each type of cancer.
**Fig. S5.** Positive correlation between gene copy numbers and expression in 10 telomerase components.
**Fig. S6.** Mutational landscapes of 9 telomerase Components.

## Data Availability

Source data downloaded from public databases are provided with this paper. Any additional information required to reanalyze the data reported in this paper is available from the corresponding authors upon reasonable request.
