# Supplementary material for: Genomic, epigenomic, and transcriptomic signatures for telomerase complex components: a pan‐cancer analysis
Source: Mol Oncol. 2022 Oct 31;17(1):150–72. doi: 10.1002/1878-0261.13324 (PMC9812836; doi:10.1002/1878-0261.13324)
Supplement: Supplementary file 1 — Fig. S1. Telomerase score (TS) and distribution of the three TS subtypes in each cancer type. Fig. S2. Correlation between telomerase score (TS) and stemness in each cancer across 33 cancer types. Fig. S3. The correlation between telomerase score (TS) and proliferation marker KI67 level in each cancer type. Fig. S4. The correlation between telomerase score (TS) and EMT score in each type of cancer. Fig. S5. Positive correlation between gene copy numbers and expression in 10 telomerase components. Fig. S6. Mutational landscapes of 9 telomerase Components. [file MOL2-17-150-s001.pdf]

Genomic, epigenomic and transcriptomic signatures for telomerase complex components: a pan-cancer analysis.

Jing Wang[1], Mingkai Dai[2], Xiangling Xing[2], Xing Wang[3], Xin Qin[4], Zhiqing Fang[4]*, Yidong Fan[4]* and Dawei Xu[2]*

Supplementary Information

Supplementary figure S1. Telomerase score (TS) and distribution of the three TS subtypes in each cancer type.

Supplementary figure S2. Correlation between telomerase score (TS) and stemness in each cancer across 33 cancer types.

Supplementary figure S3. The correlation between telomerase score (TS) and proliferation marker KI67 level in each cancer type.

Supplementary figure S4. The correlation between telomerase score (TS) and EMT score in each type of cancer.

Supplementary figure S5. Positive correlation between gene copy numbers and expression in 10 telomerase components.

Supplementary figure S6. Mutational landscapes of 9 telomerase Components

FigureS1. Telomerase scores (TS) and distribution of the three TS subtypes in each cancer type

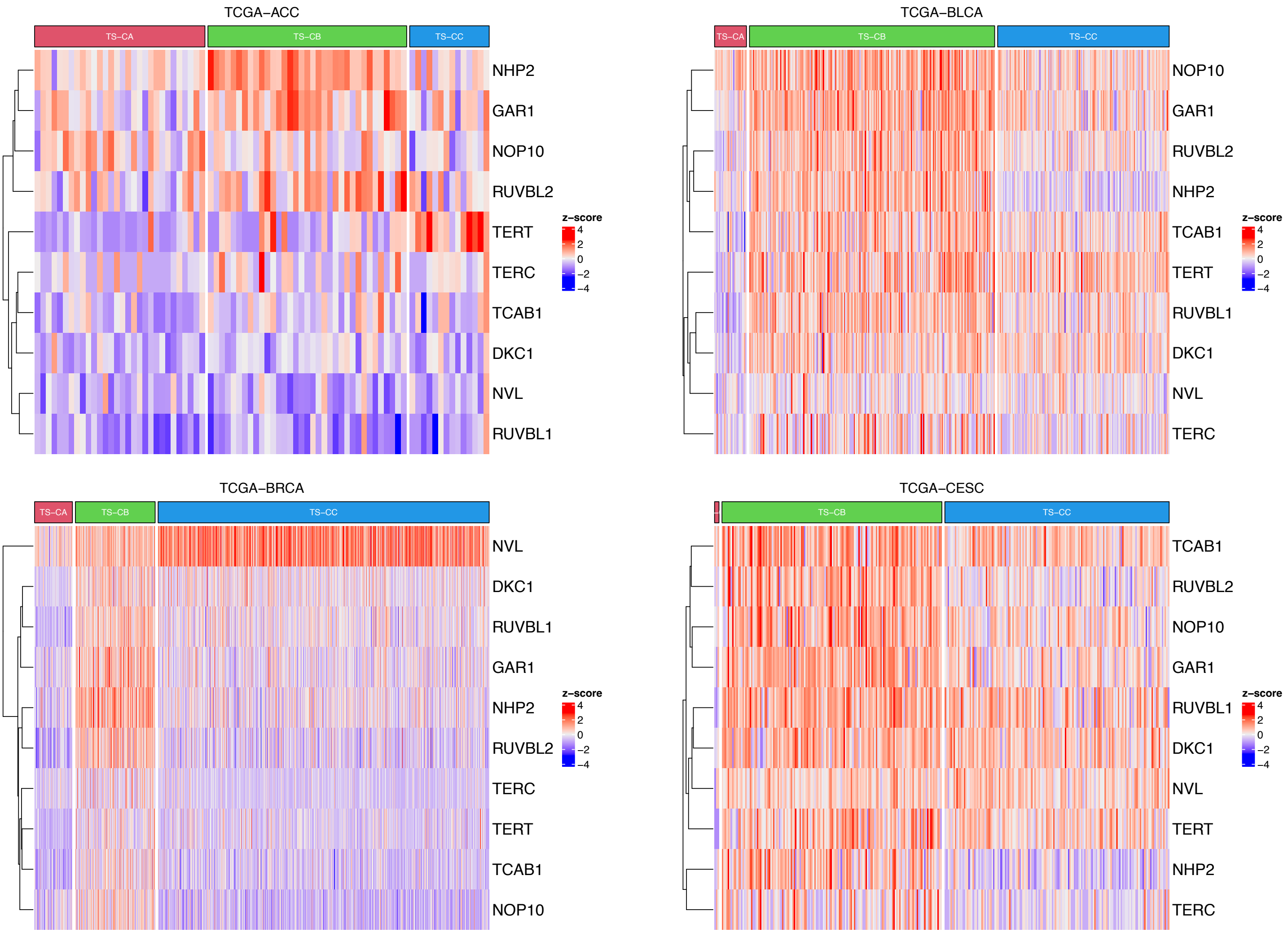

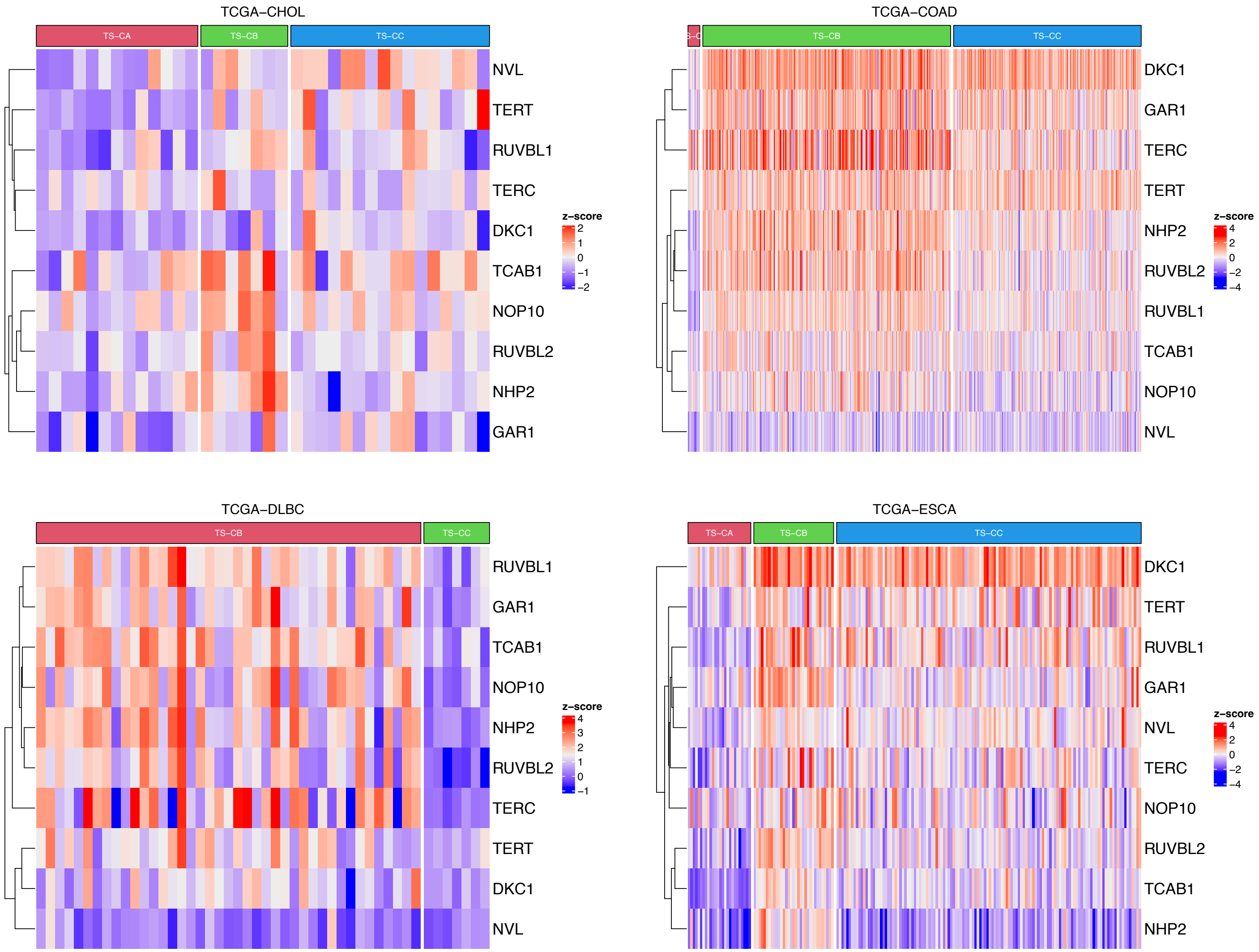
TCGA–CHOL
TS–CA
TS–CB
TS–CC
NVL
TERT
RUVBL1
TERC
DKC1
TCAB1
NOP10
RUVBL2
NHP2
GAR1
z–score
2
1
0
−1
−2
TCGA–COAD
TS–CB
TS–CC
DKC1
GAR1
TERC
TERT
NHP2
RUVBL2
RUVBL1
TCAB1
NOP10
NVL
z–score
4
2
0
−2
−4
TCGA–DLBC
TS–CB
TS–CC
RUVBL1
GAR1
TCAB1
NOP10
NHP2
RUVBL2
TERC
TERT
DKC1
NVL
z–score
4
3
2
1
0
−1
TCGA–ESCA
TS–CA
TS–CB
TS–CC
DKC1
TERT
RUVBL1
GAR1
NVL
TERC
NOP10
RUVBL2
TCAB1
NHP2
z–score
4
2
0
−2
−4

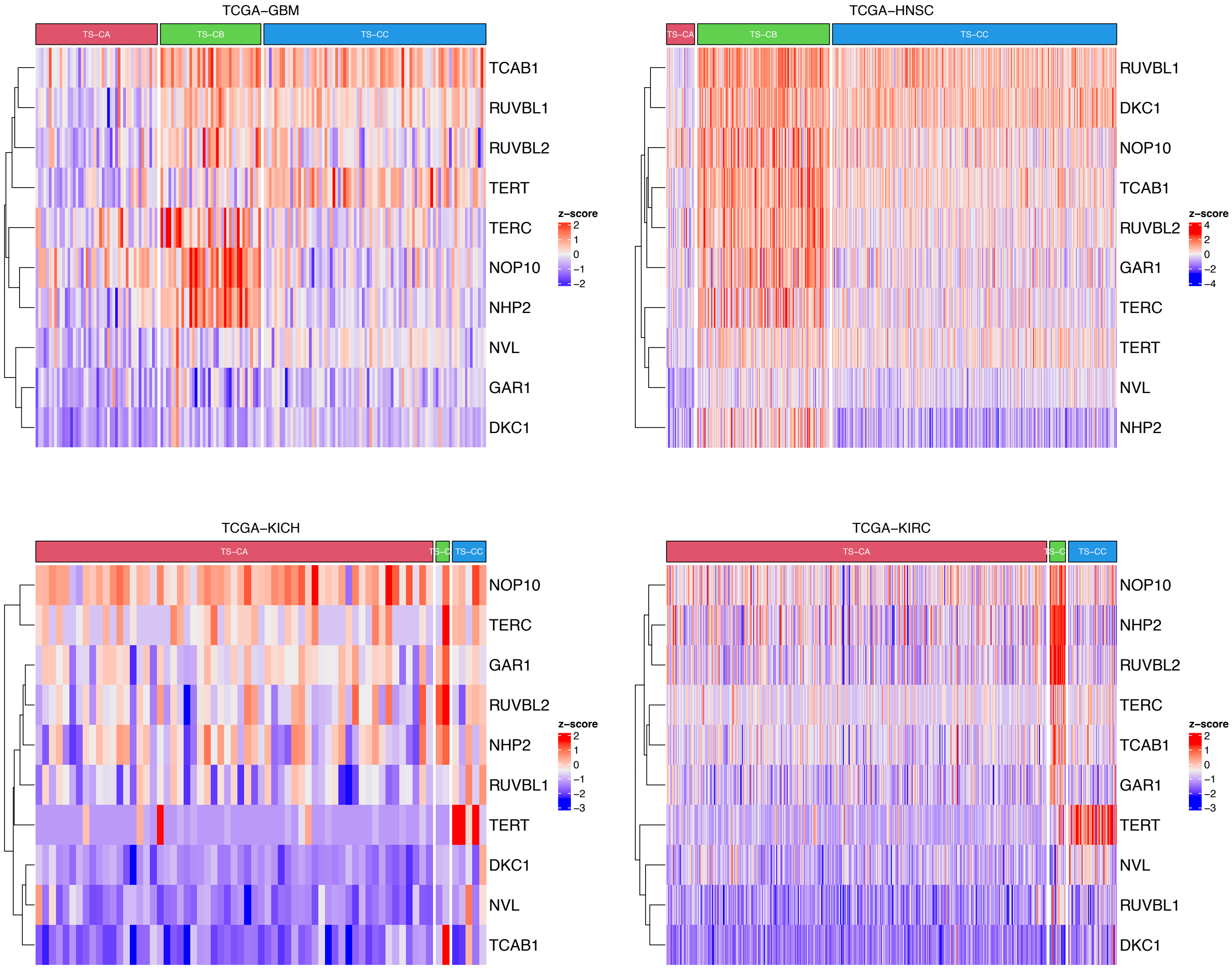
TCGA–GBM
TS–CA
TS–CB
TS–CC
TCAB1
RUVBL1
RUVBL2
TERT
TERC
NOP10
NHP2
NVL
GAR1
DKC1
z–score
2
1
0
−1
−2
TCGA–HNSC
TS–CA
TS–CB
TS–CC
RUVBL1
DKC1
NOP10
TCAB1
RUVBL2
GAR1
TERC
TERT
NVL
NHP2
z–score
4
2
0
−2
−4
TCGA–KICH
TS–CA
TS–C
TS–CC
NOP10
TERC
GAR1
RUVBL2
NHP2
RUVBL1
TERT
DKC1
NVL
TCAB1
z–score
2
1
0
−1
−2
−3
TCGA–KIRC
TS–CA
TS–C
TS–CC
NOP10
NHP2
RUVBL2
TERC
TCAB1
GAR1
TERT
NVL
RUVBL1
DKC1
z–score
2
1
0
−1
−2
−3

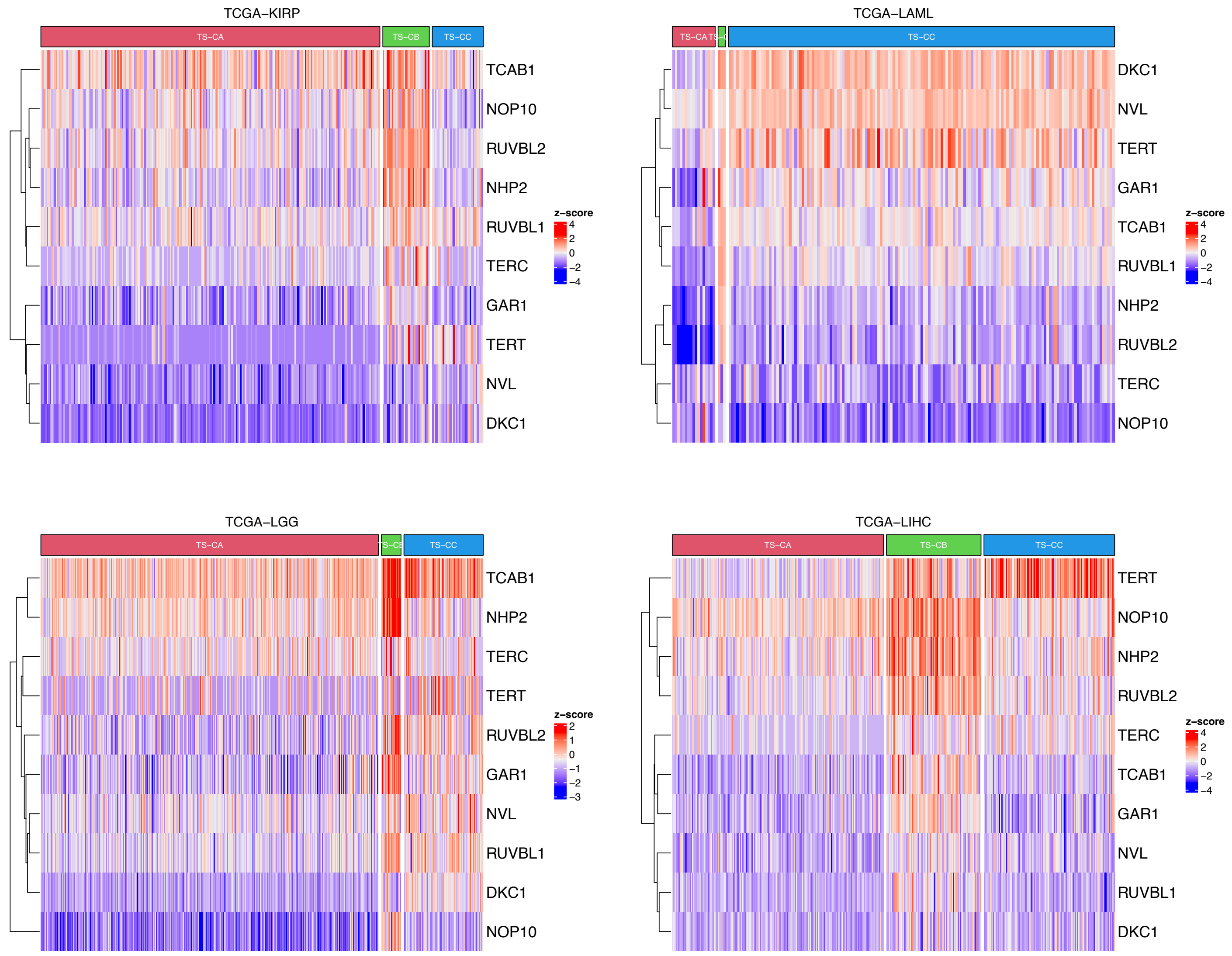
TCGA–KIRP
TS–CA
TS–CB
TS–CC
TCAB1
NOP10
RUVBL2
NHP2
RUVBL1
TERC
GAR1
TERT
NVL
DKC1
z–score
4
2
0
−2
−4
TCGA–LAML
TS–CA
TS–CC
DKC1
NVL
TERT
GAR1
TCAB1
RUVBL1
NHP2
RUVBL2
TERC
NOP10
z–score
4
2
0
−2
−4
TCGA–LGG
TS–CA
TS–CC
TCAB1
NHP2
TERC
TERT
RUVBL2
GAR1
NVL
RUVBL1
DKC1
NOP10
z–score
2
1
0
−1
−2
−3
TCGA–LIHC
TS–CA
TS–CB
TS–CC
TERT
NOP10
NHP2
RUVBL2
TERC
TCAB1
GAR1
NVL
RUVBL1
DKC1
z–score
4
2
0
−2
−4

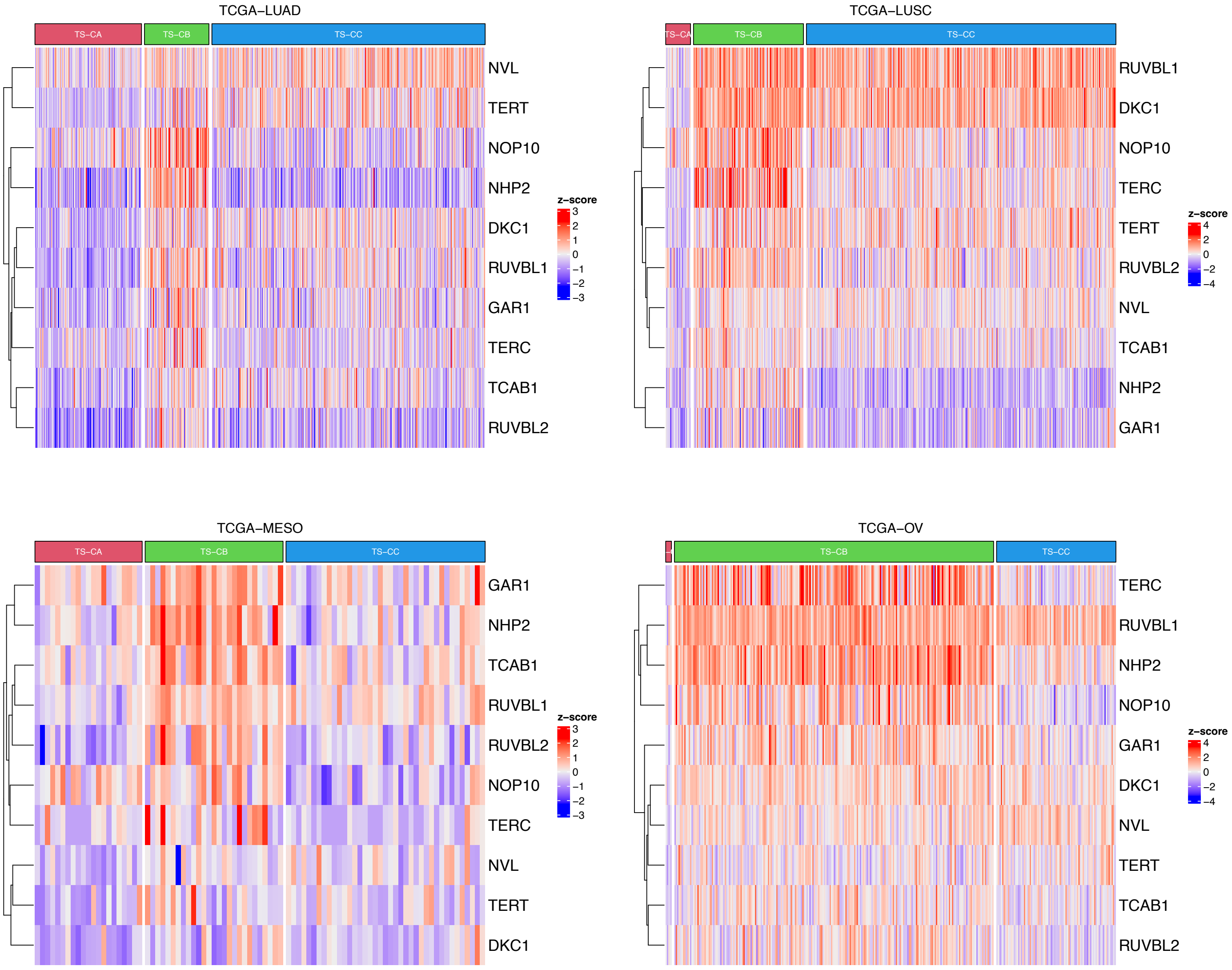
TCGA−LUAD
TS−CA
TS−CB
TS−CC
NVL
TERT
NOP10
NHP2
DKC1
RUVBL1
GAR1
TERC
TCAB1
RUVBL2
z−score
3
2
1
0
−1
−2
−3
TCGA−LUSC
TS−CA
TS−CB
TS−CC
RUVBL1
DKC1
NOP10
TERC
TERT
RUVBL2
NVL
TCAB1
NHP2
GAR1
z−score
4
2
0
−2
−4
TCGA−MESO
TS−CA
TS−CB
TS−CC
GAR1
NHP2
TCAB1
RUVBL1
RUVBL2
NOP10
TERC
NVL
TERT
DKC1
z−score
3
2
1
0
−1
−2
−3
TCGA−OV
TS−CB
TS−CC
TERC
RUVBL1
NHP2
NOP10
GAR1
DKC1
NVL
TERT
TCAB1
RUVBL2
z−score
4
2
0
−2
−4

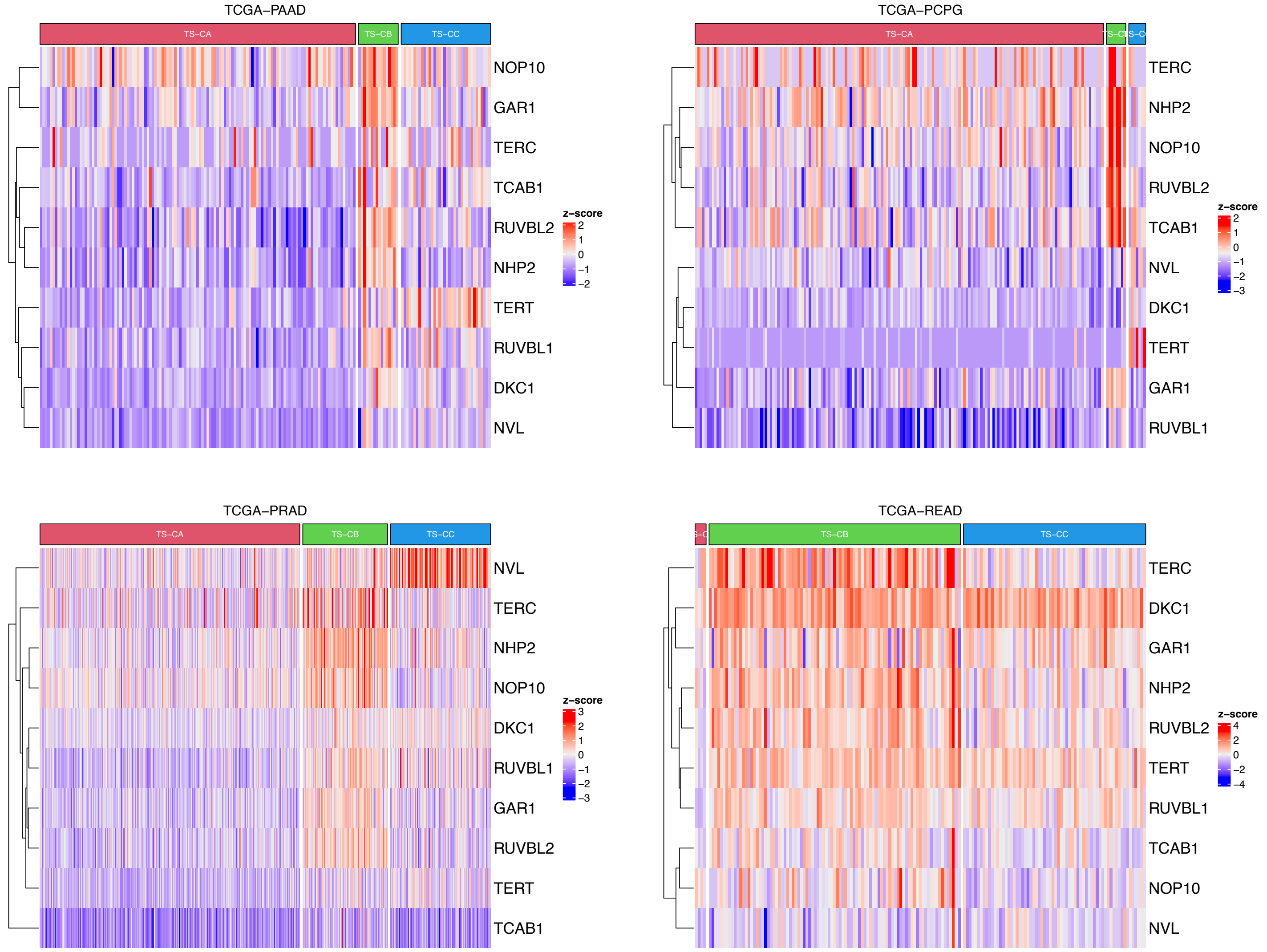
TCGA–PAAD
TS–CA
TS–CB
TS–CC
NOP10
GAR1
TERC
TCAB1
RUVBL2
NHP2
TERT
RUVBL1
DKC1
NVL
z–score
2
1
0
−1
−2
TCGA–PCPG
TS–CA
TERC
NHP2
NOP10
RUVBL2
TCAB1
NVL
DKC1
TERT
GAR1
RUVBL1
z–score
2
1
0
−1
−2
−3
TCGA–PRAD
TS–CA
TS–CB
TS–CC
NVL
TERC
NHP2
NOP10
DKC1
RUVBL1
GAR1
RUVBL2
TERT
TCAB1
z–score
3
2
1
0
−1
−2
−3
TCGA–READ
TS–CB
TS–CC
TERC
DKC1
GAR1
NHP2
RUVBL2
TERT
RUVBL1
TCAB1
NOP10
NVL
z–score
4
2
0
−2
−4

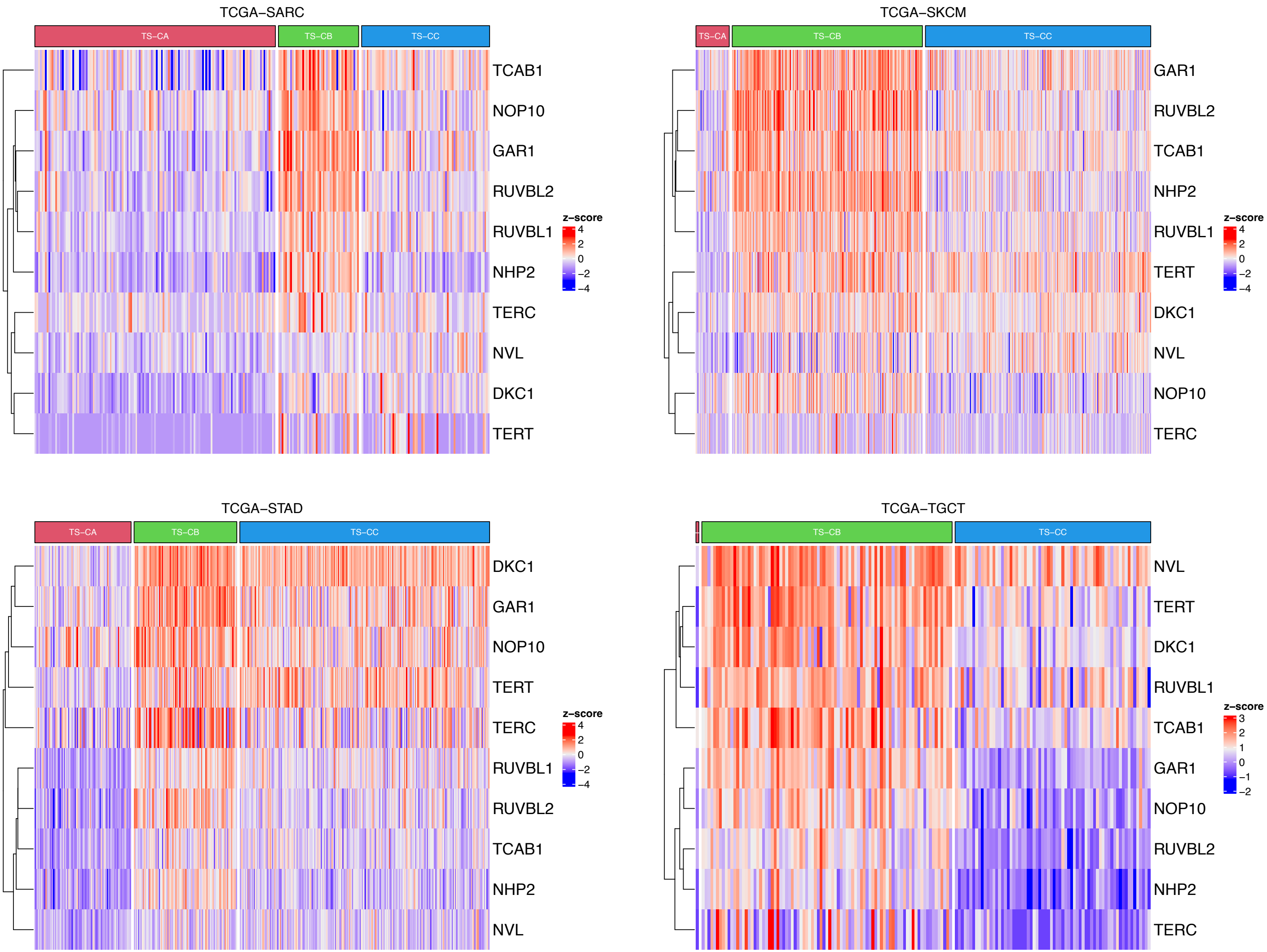
TCGA–SARC
TS–CA
TS–CB
TS–CC
TCAB1
NOP10
GAR1
RUVBL2
RUVBL1
NHP2
TERC
NVL
DKC1
TERT
z–score
4
2
0
−2
−4
TCGA–SKCM
TS–CA
TS–CB
TS–CC
GAR1
RUVBL2
TCAB1
NHP2
RUVBL1
TERT
DKC1
NVL
NOP10
TERC
z–score
4
2
0
−2
−4
TCGA–STAD
TS–CA
TS–CB
TS–CC
DKC1
GAR1
NOP10
TERT
TERC
RUVBL1
RUVBL2
TCAB1
NHP2
NVL
z–score
4
2
0
−2
−4
TCGA–TGCT
TS–CB
TS–CC
NVL
TERT
DKC1
RUVBL1
TCAB1
GAR1
NOP10
RUVBL2
NHP2
TERC
z–score
3
2
1
0
−1
−2

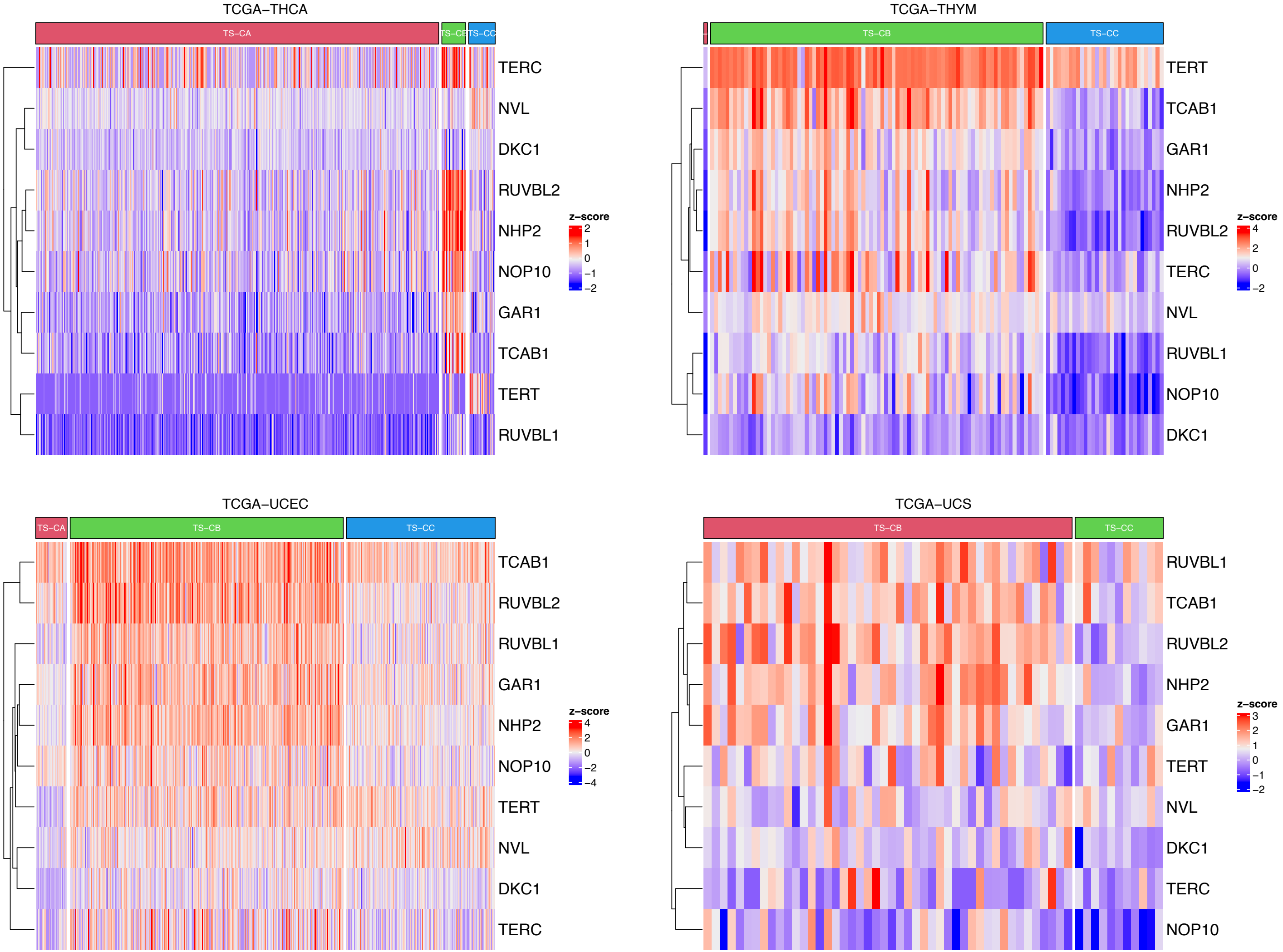

TCGA–THCA
TS–CA
TS–CC
TERC
NVL
DKC1
RUVBL2
NHP2
NOP10
GAR1
TCAB1
TERT
RUVBL1
z–score
2
1
0
−1
−2
TCGA–THYM
TS–CB
TS–CC
TERT
TCAB1
GAR1
NHP2
RUVBL2
TERC
NVL
RUVBL1
NOP10
DKC1
z–score
4
2
0
−2
TCGA–UCEC
TS–CA
TS–CB
TS–CC
TCAB1
RUVBL2
RUVBL1
GAR1
NHP2
NOP10
TERT
NVL
DKC1
TERC
z–score
4
2
0
−2
−4
TCGA–UCS
TS–CB
TS–CC
RUVBL1
TCAB1
RUVBL2
NHP2
GAR1
TERT
NVL
DKC1
TERC
NOP10
z–score
3
2
1
0
−1
−2

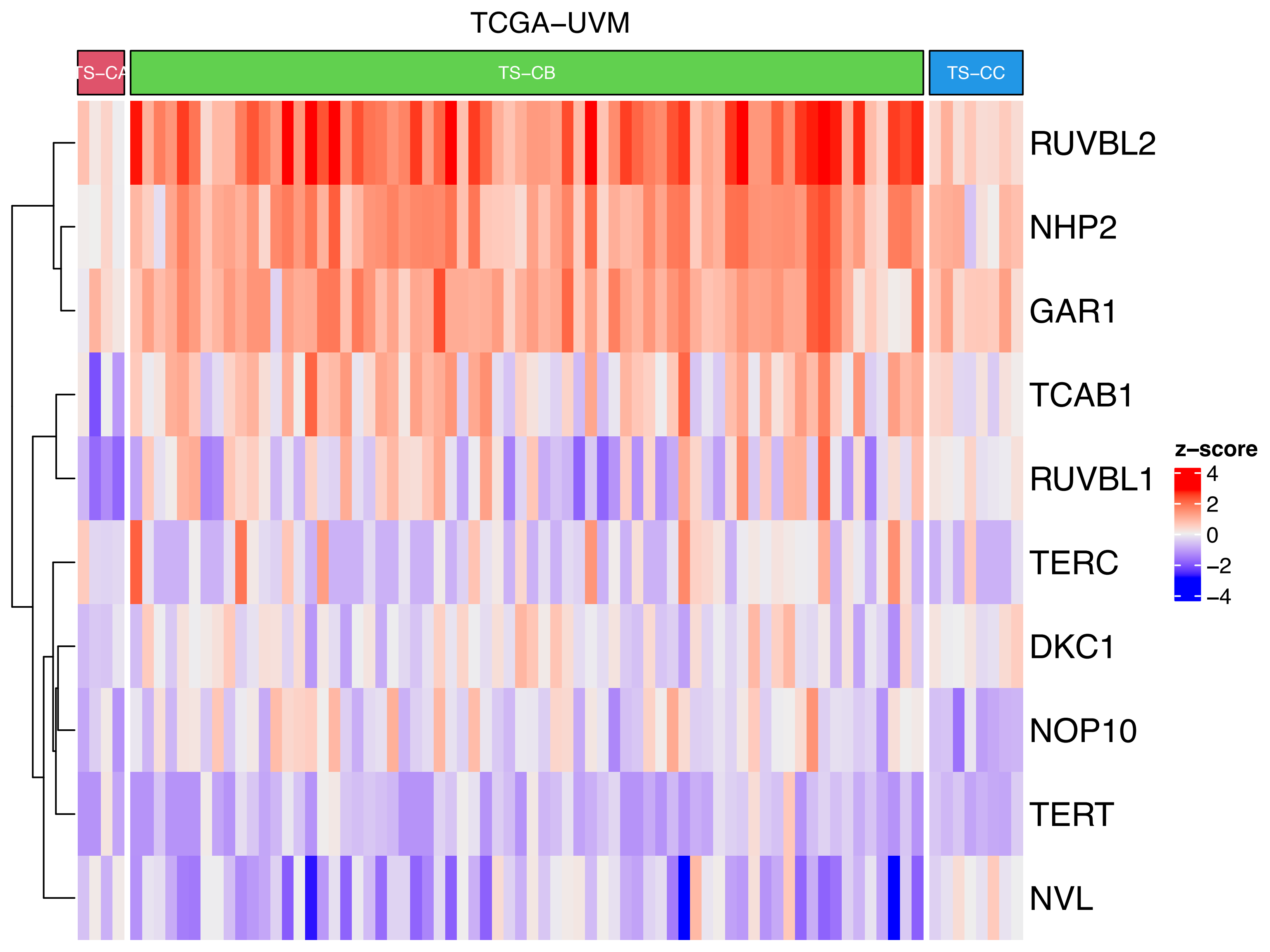
TCGA−UVM
TS−CA
TS−CB
TS−CC
RUVBL2
NHP2
GAR1
TCAB1
RUVBL1
TERC
DKC1
NOP10
TERT
NVL
z−score
4
2
0
−2
−4

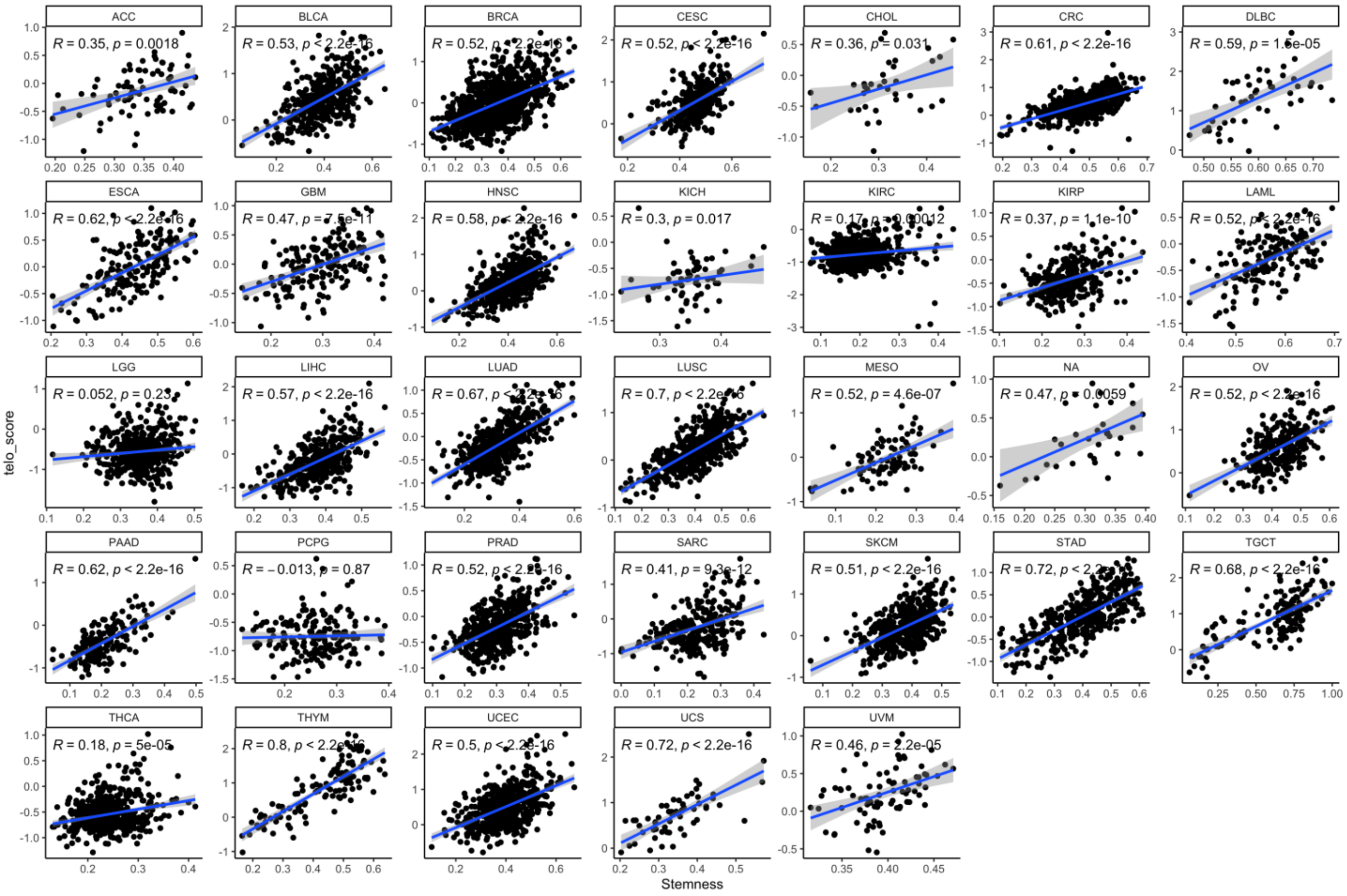

Figure S2. Correlation between telomerase score (TS) and stemness in each cancer across 33 cancer types. Positivecorrelation between TS and stemness was observed 31 of 33 cancer types except PCPG and LGG.

Figure S3. The correlation between telomerase score (TS) and proliferation marker MKI67 level in each cancer type.

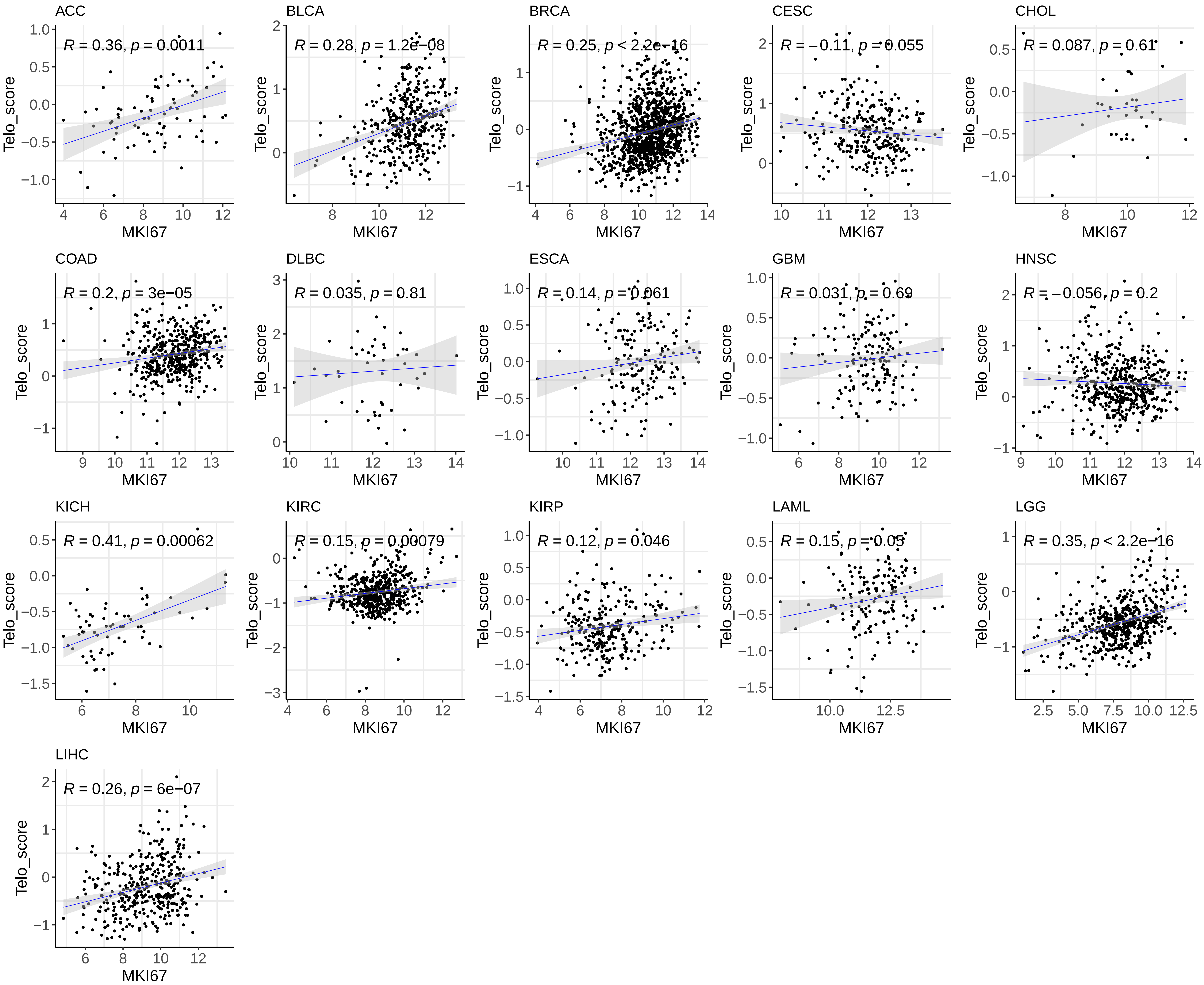

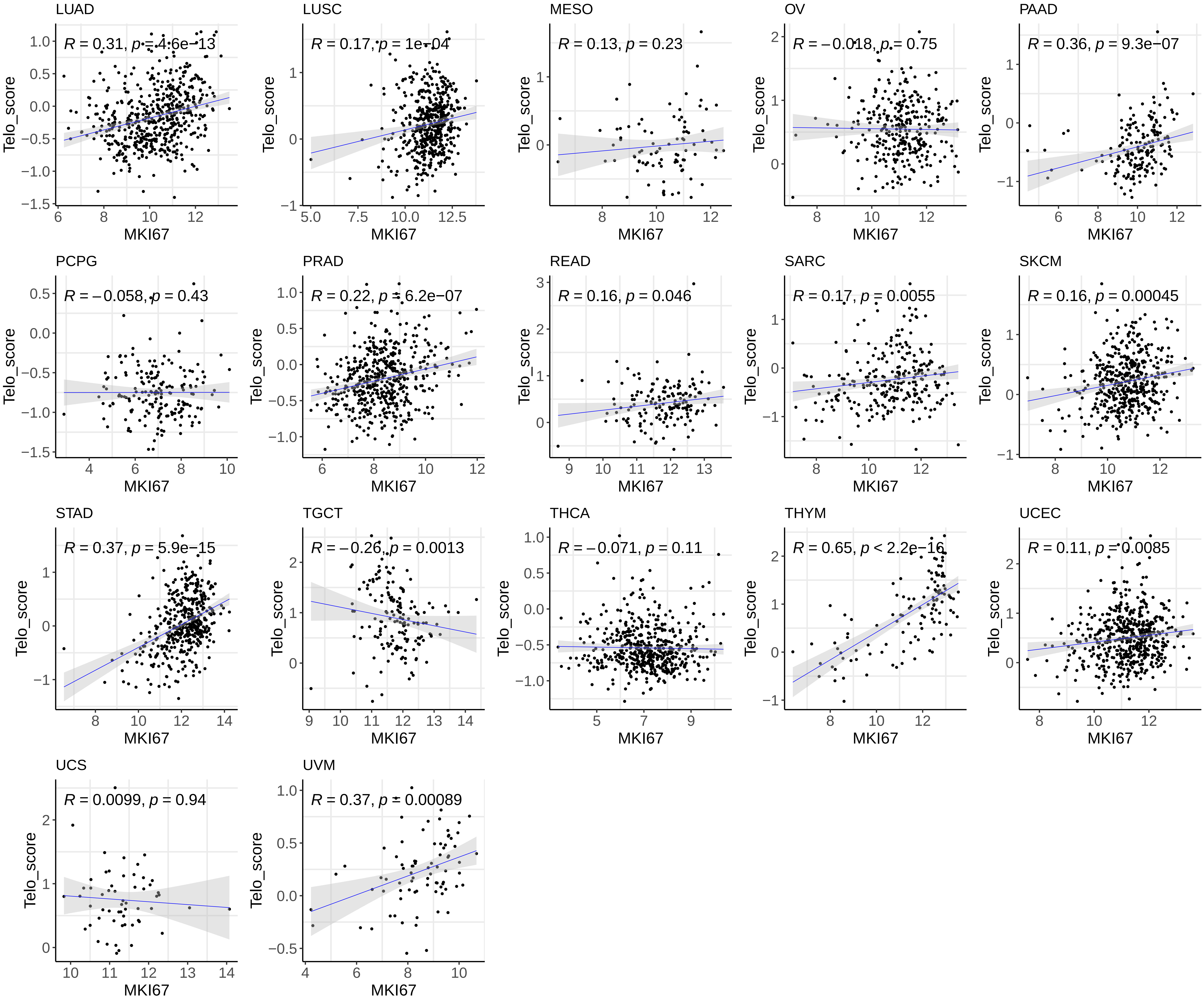
LUAD
R = 0.31, p = 4.6e−13
LUSC
R = 0.17, p = 1e−04
MESO
R = 0.13, p = 0.23
OV
R = −0.018, p = 0.75
PAAD
R = 0.36, p = 9.3e−07
PCPG
R = −0.058, p = 0.43
PRAD
R = 0.22, p = 6.2e−07
READ
R = 0.16, p = 0.046
SARC
R = 0.17, p = 0.0055
SKCM
R = 0.16, p = 0.00045
STAD
R = 0.37, p = 5.9e−15
TGCT
R = −0.26, p = 0.0013
THCA
R = −0.071, p = 0.11
THYM
R = 0.65, p < 2.2e−16
UCEC
R = 0.11, p = 0.0085
UCS
R = 0.0099, p = 0.94
UVM
R = 0.37, p = 0.00089
Telo_score
MKI67

Figure S4. The correlation between telomerase score (TS) and EMT score in each type of cancer

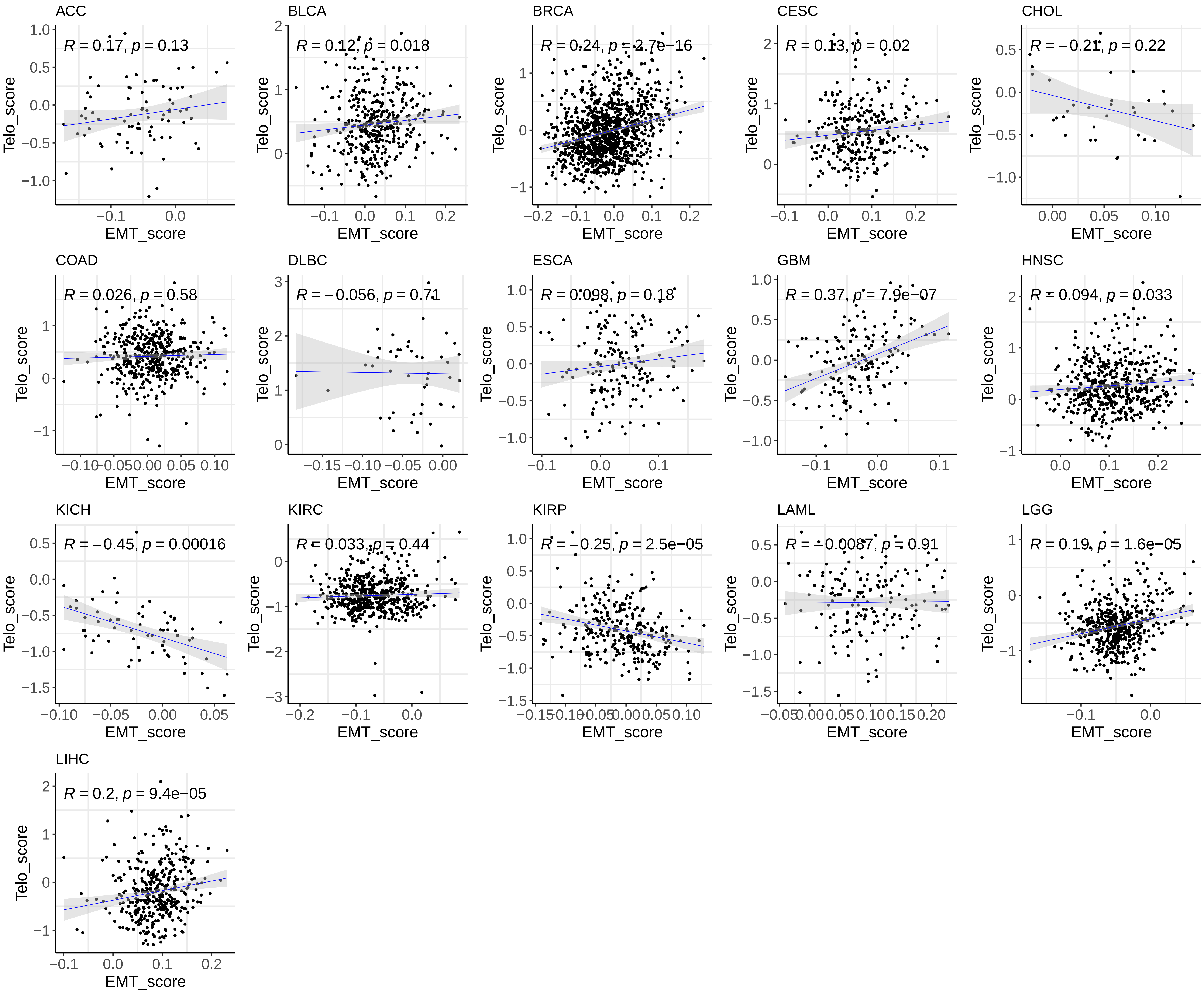

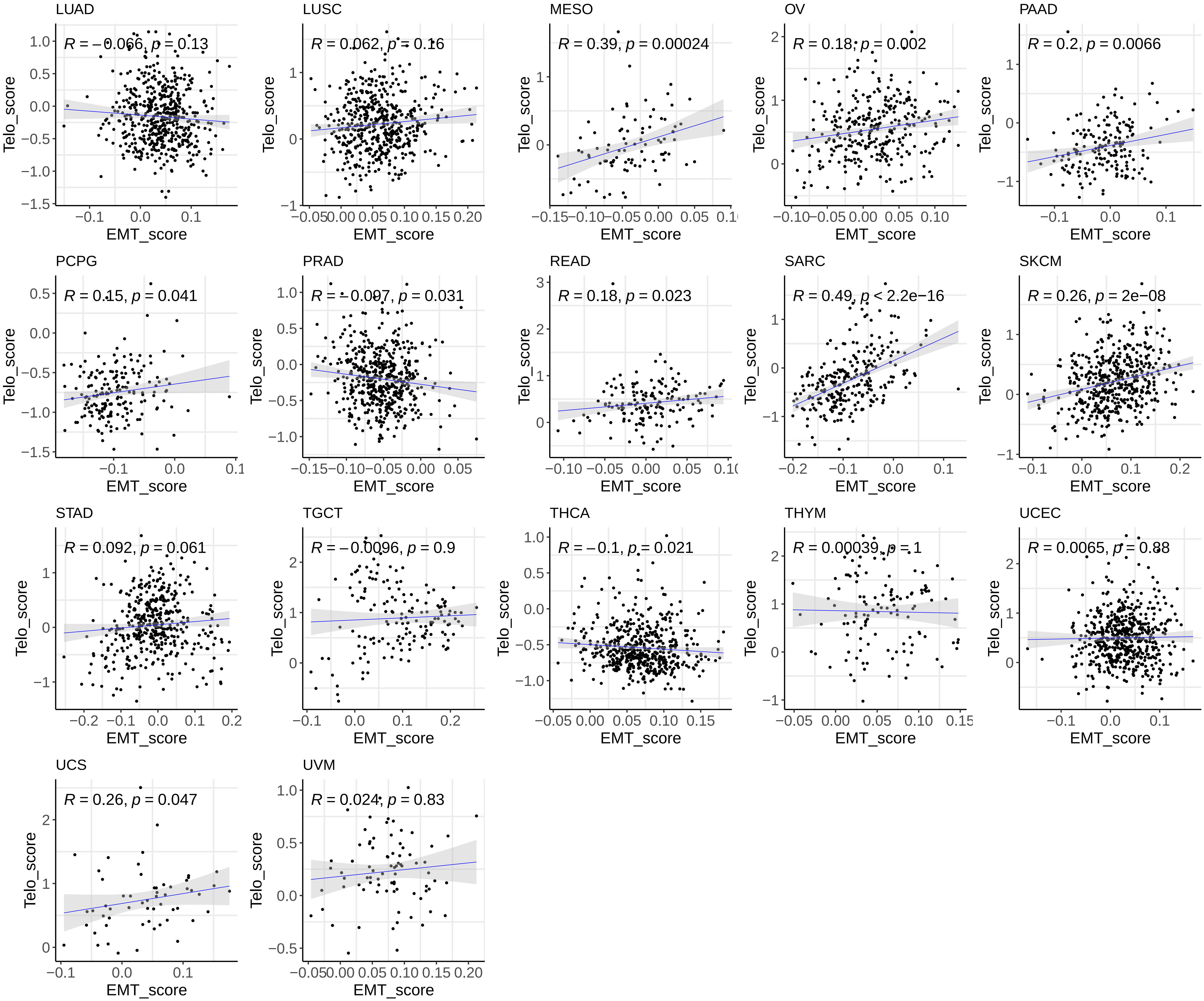
LUAD
R = −0.066, p = 0.13
LUSC
R = 0.062, p = 0.16
MESO
R = 0.39, p = 0.00024
OV
R = 0.18, p = 0.002
PAAD
R = 0.2, p = 0.0066
PCPG
R = 0.15, p = 0.041
PRAD
R = −0.097, p = 0.031
READ
R = 0.18, p = 0.023
SARC
R = 0.49, p < 2.2e−16
SKCM
R = 0.26, p = 2e−08
STAD
R = 0.092, p = 0.061
TGCT
R = −0.0096, p = 0.9
THCA
R = −0.1, p = 0.021
THYM
R = 0.00039, p = 1
UCEC
R = 0.0065, p = 0.88
UCS
R = 0.26, p = 0.047
UVM
R = 0.024, p = 0.83
Telo_score
EMT_score

Figure S5. Correlation between gene copy numbers and expression of ten telomerase components in pan-cancers

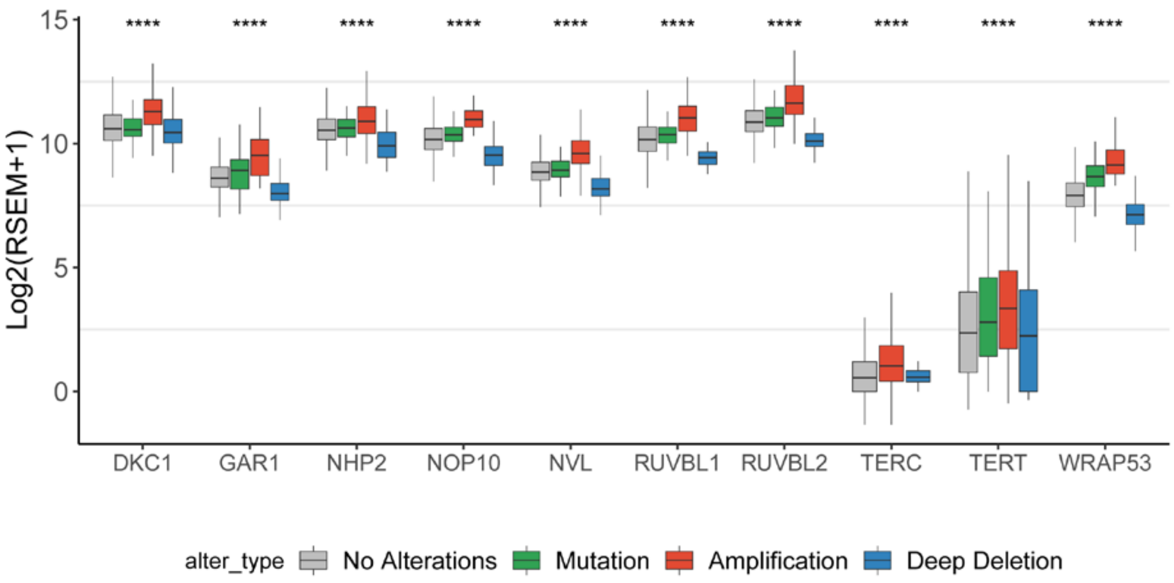

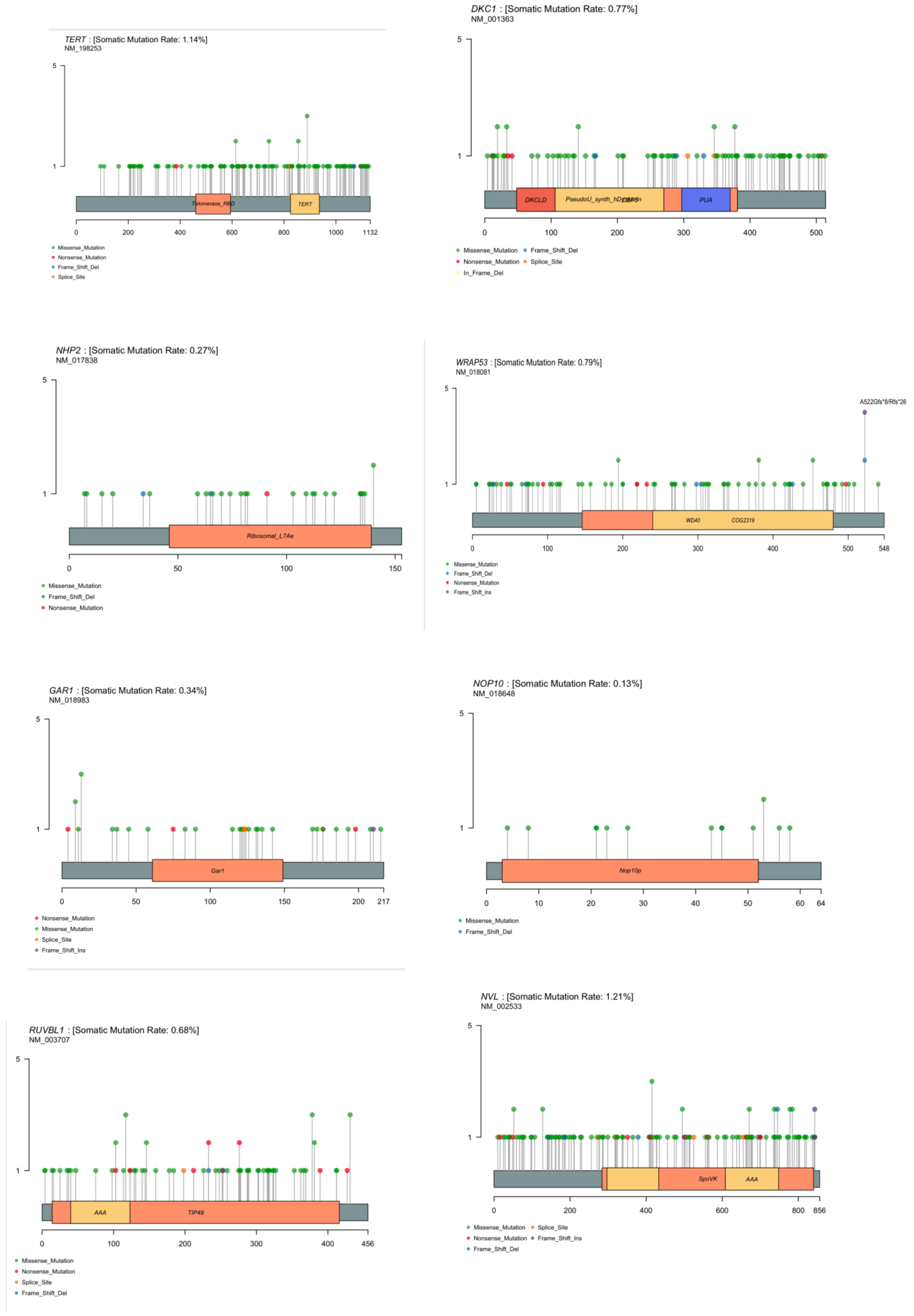

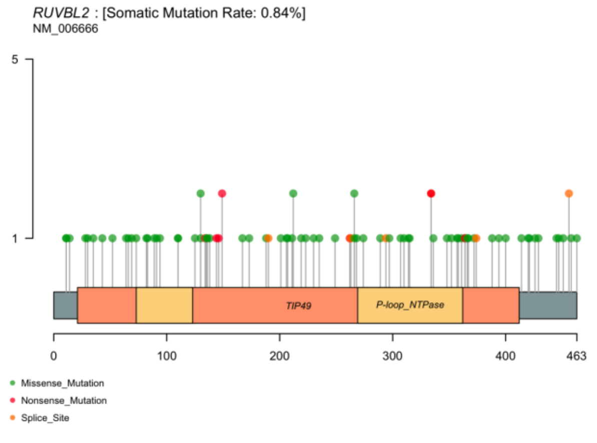

Figure S6. Mutational landscapes of 9 telomerase Components.
